# p38b and JAK-STAT signaling protect against Invertebrate iridescent virus 6 infection in *Drosophila*

**DOI:** 10.1371/journal.ppat.1007020

**Published:** 2018-05-10

**Authors:** Cara West, Neal Silverman

**Affiliations:** Program in Innate Immunity, Division of Infectious Diseases and Immunology, Department of Medicine, University of Massachusetts Medical School, Worcester, Massachusetts, United States of America; University of Pennsylvania School of Medicine, UNITED STATES

## Abstract

The fruit fly *Drosophila melanogaster* is a powerful model system for the study of innate immunity in vector insects as well as mammals. For vector insects, it is particularly important to understand all aspects of their antiviral immune defenses, which could eventually be harnessed to control the transmission of human pathogenic viruses. The immune responses controlling RNA viruses in insects have been extensively studied, but the response to DNA virus infections is poorly characterized. Here, we report that infection of *Drosophila* with the DNA virus Invertebrate iridescent Virus 6 (IIV-6) triggers JAK-STAT signaling and the robust expression of the *Turandots*, a gene family encoding small secreted proteins. To drive JAK-STAT signaling, IIV-6 infection more immediately induced expression of the *unpaireds*, a family of IL-6-related cytokine genes, via a pathway that required one of the three *Drosophila* p38 homologs, p38b. In fact, both *Stat92E* and *p38b* were required for the survival of IIV-6 infected flies. In addition, *in vitro* induction of the *unpaireds* required an NADPH-oxidase, and *in vivo* studies demonstrated *Nox* was required for induction of *TotA*. These results argue that ROS production, triggered by IIV-6 infection, leads to p38b activation and *unpaired* expression, and subsequent JAK-STAT signaling, which ultimately protects the fly from IIV-6 infection.

## Author summary

Mosquitoes and other biting insects transmit many harmful pathogens to humans, including parasites and viruses. In order to better protect humans from these diseases, we must gain a more complete understanding of how insects successfully—or unsuccessfully—combat these infections. While we know a great deal regarding how insects combat RNA viruses, we know little about their immune response to DNA virus infections. Studies of DNA virus infections may reveal novel immune mechanisms, which could be uniquely effective against DNA virus infections or could be broadly effective against many viruses. In this study, we utilized an invertebrate DNA virus, IIV-6, infection model with the fruit fly *Drosophila melanogaster*, and found that virus infection activated several innate immune signaling pathways, which help protect the animal against this virus. Eventually, a more complete understanding of the antiviral responses of insects may be useful to restrict virus infections of disease transmitting insects.

## Introduction

Like all multicellular organisms, insects face a constant threat of infection from a wide array of microorganisms, including viral, bacterial, and fungal pathogens. Insects combat these infections with both static and inducible defenses, including a chitinous exoskeleton, circulating phagocytes and the induction of host defense genes, such as antimicrobial peptides [1]. For example, *Drosophila melanogaster* respond to bacterial and fungal infections through two conserved NF-κB signaling pathways, Toll and Imd, that drive the production of antimicrobial peptides and other inducible host defense molecules. Toll and Imd pathways are homologous to the TLR-MyD88 and TLR-TRIF signaling pathways in mammals, respectively. Unlike mammals, the *Drosophila* NF-κB pathways seem to play a limited role in response to invertebrate viral infections [2–5].

Insects, especially mosquitoes, are major vectors of arboviral diseases, and characterizing the pathways that they utilize to combat viral infections is necessary to gain a complete understanding of disease transmission. *Drosophila*, also a dipteran insect, has served as a productive model for studying insect antiviral immunity [6]. In *Drosophila*, the most intensely studied antiviral pathway is the siRNA response. The siRNA response is triggered when viral dsRNA intermediates, either derived directly from the viral genome or produced as an intermediate during replication or transcription, are recognized by Dicer-2, which processes these dsRNAs into 21 base-pair fragments and loads them onto Argonaute-2. This complex, termed an RNA-Induced Silencing Complex (RISC), is then able to destroy its complementary target sequence. This mechanism is a potent antiviral defense against RNA viruses and, in some circumstances, against DNA viruses [7, 8]. In addition, it has been suggested that Dicer-2, following the recognition of viral dsRNA, can trigger a signaling pathway that induces the transcription of the antiviral gene Vago [9]. While antiviral effects of *Vago* have only been shown upon Drosophila C virus (DCV) infection of flies, mosquito *Vago* was induced upon flavivirus infections of Culicine cell lines and animals and was suggested to be antiviral [10, 11].

On the other hand, mammalian antiviral defenses are triggered after recognition of an array of pathogen-associated and damage-associated molecular patterns (PAMPs and DAMPs), including but not limited to dsRNA. For example, various RNA species are recognized in the endosome by TLR3, 7 & 8, or in the cytosol by RIG-I or MDA-5. In addition, numerous viral proteins are recognized directly by various TLRs [12], and viral-induced damage often leads to induction of inflammasome activation and pyroptosis or necroptosis. Inflammasome activation results in the production of proinflammatory cytokines to promote recruitment of effector cells to the site of infection, while pyroptosis can act to restrict viral infection by killing the infected cell and thereby limiting viral replication [13].

Compared to mammalian systems, the extent to which the invertebrate immune system recognizes PAMPs and DAMPs, beyond viral dsRNA, is less well-studied. Examples from *Drosophila* include Toll-7, which was demonstrated to sense vesicular stomatitis virus (VSV) and Rift Valley fever virus (RVFV), and activate autophagy as an antiviral defense [14, 15]. Additionally, JAK-STAT signaling has been shown to induce *vir-1* in response to DCV or cricket paralysis virus (CrPV), although the mechanism by which these viruses activate JAK-STAT signaling is unknown [8]. Imd signaling in the gut, triggered by commensals, is required for the activation of the ERK pathway, which protects against several enteric RNA viruses [5]. This commensal-mediated priming may explain the previously reported activity of Imd and Toll pathways in protecting against other viral infections [2–4, 16]. A study examining the origins of cGAS-STING signaling—an important cytosolic DNA sensing pathway in mammals, leading to Type I interferon production—concluded that cGAS homologs in insects lack regions required for DNA binding [17]. Thus, the role of the cGAS homologs in insects remains unclear.

In fact, the pathways responding to DNA virus infections in *Drosophila* have not been characterized. Insect DNA viruses, such as baculoviruses, the invertebrate iridescent viruses, and polydnaviruses are large, complex viruses ranging from 50 to more than 200 genes. These viral genes include many immunomodulators and other proteins that manipulate the cellular environment to facilitate replication [18]. These large DNA viruses and their myriad of host targeting and manipulating factors suggest that many host immune response pathways, beyond RNAi, interfere with viral replication.

Here, we report that *Drosophila* infection with the DNA virus Invertebrate iridescent virus 6 (IIV-6) induces a p38b-dependent pathway that activates JAK-STAT signaling and the robust transcriptional induction of a class of small secreted proteins known as the Turandots. Activation of this pathway requires the production of reactive oxygen species, perhaps indicative of a response to damage. Moreover, two key components of this pathway, p38b and Stat92E, are essential for host defense against IIV-6 infection.

## Results

### Turandots are induced by IIV-6 infection

**Fig 1:**
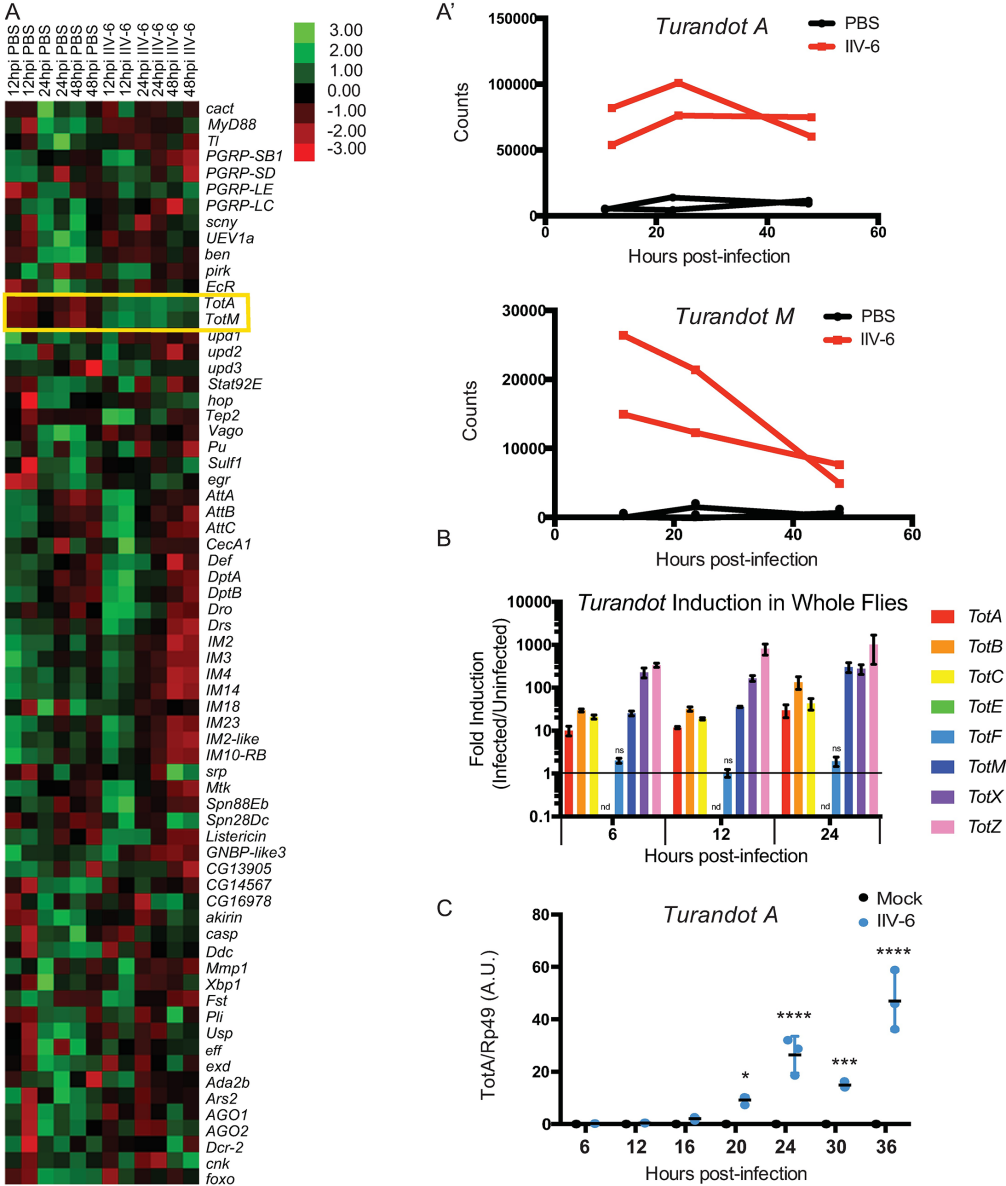
**Turandot genes are expressed upon IIV-6 Infection A)** Heatmap of mRNA levels for selected immune-related genes following IIV-6 infection of adult *w*^*1118*^ flies for 12, 24 and 48 hours assayed in duplicate by NanoString nCounter. RNA was isolated from PBS-injected flies at the same time points. Each data point is a biologically independent sample. **A’)** Detailed comparison of mRNA levels for *Tot A* or *Tot M* from nCounter data. **B)** Fold induction of all eight *Tots* from *w*^*1118*^ flies infected with IIV-6, relative to PBS injected controls, at 6, 12, or 24 hours, quantified by qRT-PCR. n = 3, error bars represent SEM and statistical significance determined by Multiple t-tests with correction for multiple comparisons using the Holm-Sidak method. By this analysis, all *Turandots* were significantly induced with p values between 0.05 and 0.0003 at all time-points with the exception of *TotF* (ns) and *TotE*, which was undetectable (nd). **C)** S2* cells were infected with IIV-6 and *TotA* expression was assayed by qRT-PCR at the indicated time points. Significance was determined by two-way ANOVA and Sidak’s multiple comparisons test, comparing the infected sample to its time-matched uninfected control. * p < 0.05; *** p < 0.001; and **** p < 0.0001 Error bars indicate standard deviation and black bars indicate the mean. A.U., Arbitrary Units.

Given the paucity of data on the insect response to DNA virus infection, we sought to identify the immune-related genes upregulated upon IIV-6 infection of adult flies. Male flies were injected with PBS (vehicle control) or IIV-6 at 1x10^4^ TCID50 for 12, 24, or 48 hours, RNA was isolated and analyzed by NanoString nCounter Analysis, with a codeset probing 139 immune-related genes (Fig 1A). Two members of the *Turandot* (*Tot*) family, *TotA* and *TotM* were strongly upregulated at all time points in the IIV-6 infected samples compared to PBS-injected controls (Fig 1A and 1A’). These genes are part of a family of eight closely related, rapidly evolving genes that are induced by a variety of stressors including bacterial infection, heat shock, mechanical pressure, and UV-exposure [19, 20]. The *Tots* encode for small secreted proteins that have no known function [19, 21]. As the NanoString codeset included only two *Tot* genes, we used qRT-PCR to examine the expression of all eight *Tot* genes following IIV-6 infection. Six *Tot* genes were induced 10–1000 fold, compared to the PBS injected controls, 6 to 24 hours following IIV-6 infection (Fig 1B). The two *Tot* genes not up-regulated, *TotE* and *TotF*, are clustered together on Chromosome 2, suggesting that these two closely related Tots may respond to a different set of stimuli. Note, *TotE* was undetectable, while *TotF* was detected but unchanged by IIV-6 infection. *Drosophila* S2* cells also induced *TotA*, peaking between 24–36 hours after IIV-6 infection, while in mock-treated controls *TotA* expression remained at baseline levels (Fig 1C).

**Fig 2:**
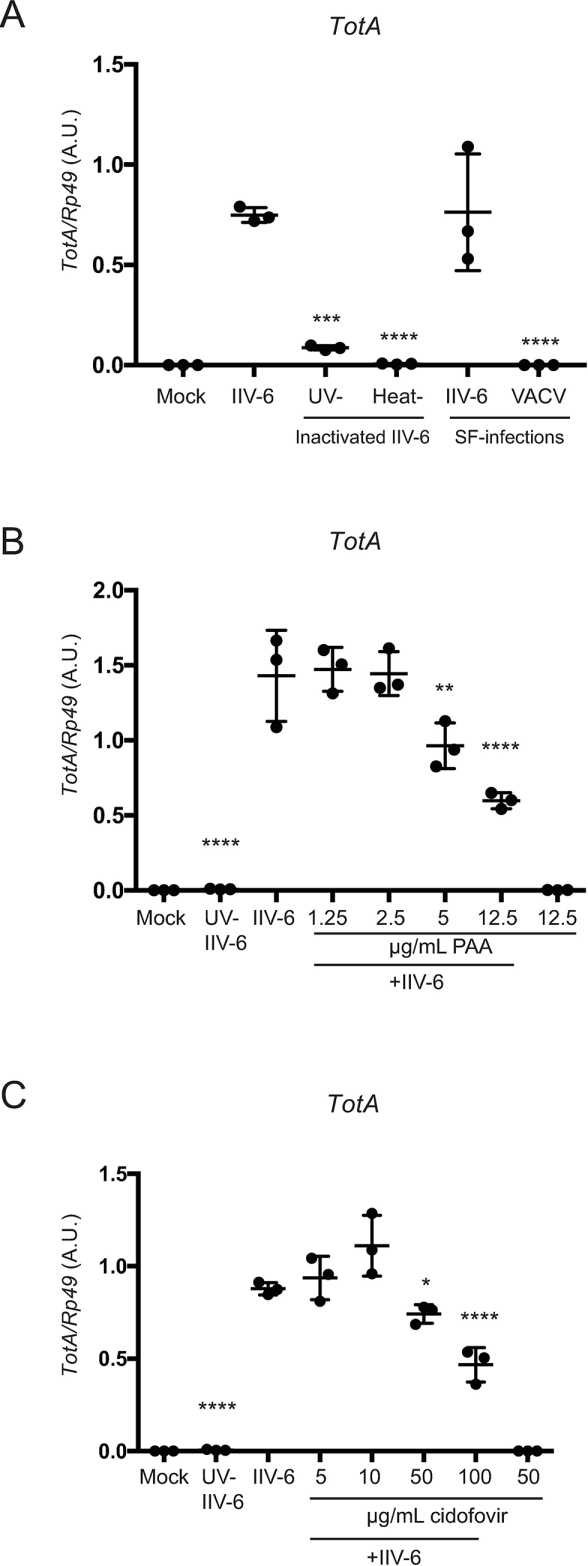
Viral replication is required for IIV-6 induced *turandot* expression. **A)** S2* cells were infected with heat- or UV-inactivated IIV-6 or infected with VACV or IIV-6 in serum-free (SF) media, and *TotA* induction was assayed by qRT-PCR. **B, C)** S2* cells were treated with the viral polymerase inhibitors **B)** phosphonoacetic acid (PAA) or **C)** cidofovir at the indicated concentrations for one hour prior to IIV-6 infection. For all panels, *TotA* induction was assayed at 24 hours post-infection by qRT-PCR. Significance, compared to IIV-6 infected samples without treatment or drugs, was determined by one-way ANOVA and Sidak’s multiple comparisons test (* p < 0.05; *** p < 0.001; and **** p < 0.0001). Error bars indicate standard deviation and black bars indicate the mean. A.U., Arbitrary Units.

To begin to dissect the mechanisms that lead from DNA virus infection to *Tot* gene induction, we tested the requirement for live virus infection and viral replication in this process. IIV-6 was heat- or UV-inactivated, which typically creates virus that can attach, enter cells, and possibly deliver damaged nucleic acids or protein but is not replicative [22]. These inactivated viruses were then used to stimulate S2* cells (Fig 2A). Both UV-inactivation and heat treatment significantly reduced *TotA* induction, to levels near baseline. In addition, infection with another DNA virus, Vaccinia virus (VACV), which is known to abortively infect S2 cells [23, 24], failed to induce *TotA* expression (Fig 2A). Consistent with this observation, pretreatment of S2* cells with viral DNA polymerase inhibitors, phosphonoacetic acid (PAA) or cidofovir, also resulted in significantly diminished IIV-6 triggered *TotA* induction (Fig 2B and 2C). These results suggest that the presence of viral DNA alone is not sufficient to trigger *Tot* induction, in contrast to mammalian systems where cytosolic DNA triggers a robust cytokine response and triggers inflammasome activation [25]. Studies with the polymerase inhibitors PAA and cidofovir further argue that virus entry and expression of early gene transcripts are also not sufficient for this response. Together, these data indicate that active viral replication (or the processes downstream of viral replication) is required for IIV-6 induced *TotA* induction.

### Turandot induction is JAK-STAT dependent

**Fig 3:**
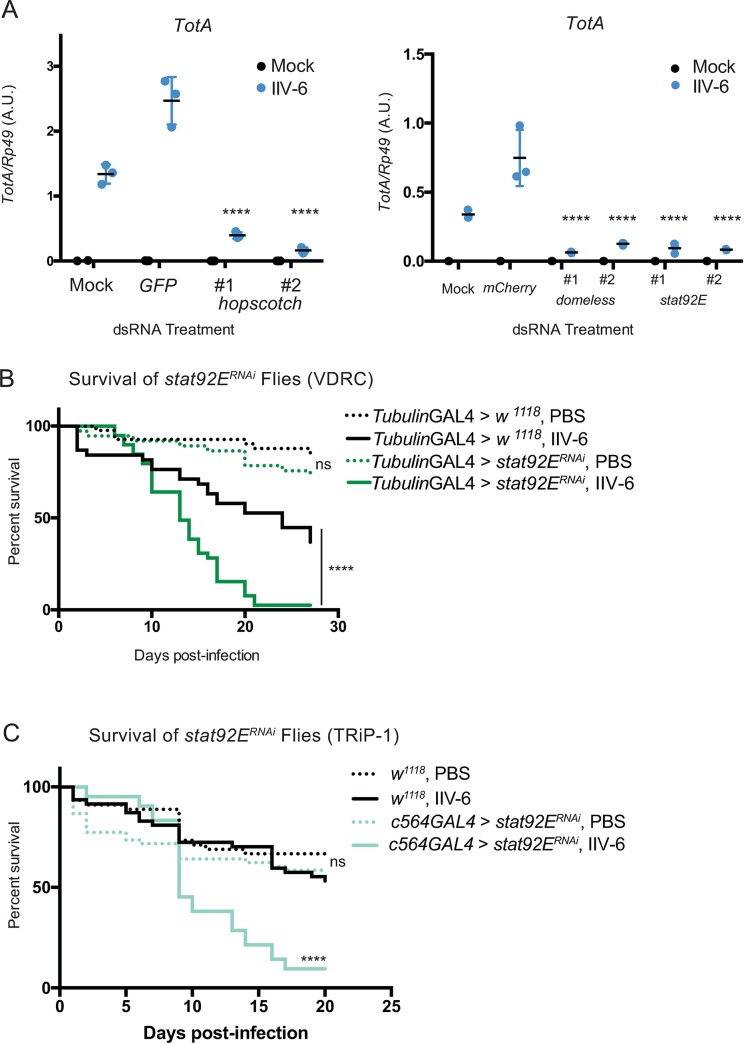
**JAK-STAT signaling is required for IIV-6-induced *turandot* expression and survival from virus infection A)** S2* cells were transfected with dsRNA targeting *hopscotch*, *domeless*, or *Stat92E*, and 48 hours later were infected with IIV-6 for 24 hours. RNA was then isolated and *TotA* induction was quantified by qRT-PCR. Data shown are from three biologically independent assays. Two non-overlapping dsRNAs were used to target each gene. Error bars indicate standard deviation and black bars indicate mean. Statistical analysis was performed comparing control dsRNA (*GFP* or *mCherry*) transfected cells to target gene knockdowns by two-way ANOVA with corrections for multiple comparisons using the Holm-Sidak method (**** p <0.0001). A.U., Arbitrary Units. **B)** Kaplan-Meier curves showing survival of *Stat92E*^*RNAi*^ expressing (*UAS-Stat92E*^*RNAi*^ (VDRC)*x tubulin-Gal4)* flies (green lines) or control (*w*^*1118*^ *x tubulin-Gal4)* flies (black lines) following infection with IIV-6 (solid lines) or injection with PBS (dotted lines). **C)** Kaplan-Meier curves showing survival of *Stat92E*^*RNAi*^ expressing (*UAS-Stat92E*^*RNAi*^ (TRiP-1)*x c564-Gal4)* flies (green lines) or control (*w*^*1118*^) flies (black lines) following infection with IIV-6 (solid lines) or injection with PBS (dotted lines). Survival assays utilized at least 50 animals per treatment in each trial and statistical significance was determined by Log-rank (Mantel-Cox) test, comparing IIV-6 infected RNAi lines to IIV-6 infected control animals, or PBS-injected RNAi lines to PBS-injected controls. ****p <0.0001.

Previous studies demonstrated that the JAK-STAT signaling pathway is responsible for inducing *Tot* expression after Gram-negative bacterial infection [26]. To test whether JAK-STAT signaling is also required for IIV-6-induced *Tot* expression, *domeless*, *hopscotch*, *and Stat92E*, the sole *Drosophila* homologs for the gp130 receptor, JAK, and STAT, respectively, were targeted by RNAi in S2* cells. Knockdown of any of these genes led to a significant decrease in *TotA* induction (Fig 3A, knock-down efficiencies S1A–S1C Fig). We then asked whether the JAK-STAT pathway had any effect on survival following IIV-6 infection. *Stat92E* was ubiquitously knocked down using the *tubulin-GAL4* driver and these flies were challenged with 10^4^ TCID50 of IIV-6. The *Stat92E* knockdown flies exhibited significantly increased lethality compared to the control strain (progeny of *w*^*1118*^ x *tubulin-GAL4*) after virus infection (Fig 3B, additional trials S2 Fig, statistical analysis for all survival assays can be found in S2 Table). On the other hand, both control and *Stat92E* knockdown lines tolerated the control PBS injection to a similar degree. This was confirmed using additional RNAi lines (Figs 3C and S1E), and all lines had significant knock-down efficiencies (S1D Fig). These results demonstrate that IIV-6 induced *Tot* expression is controlled by the JAK-STAT pathway, and this pathway is critical for survival following infection.

**Fig 4:**
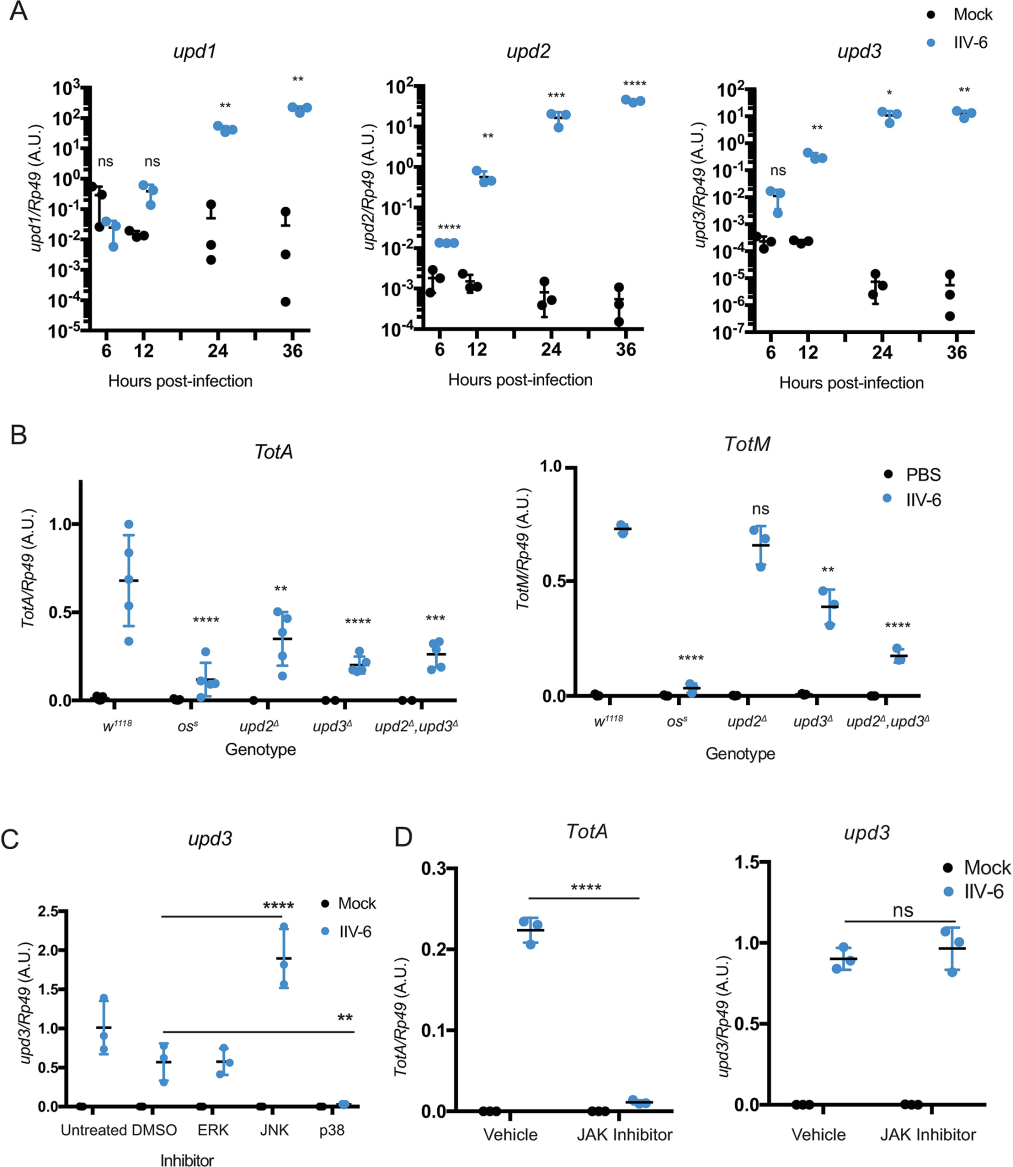
**p38-dependent IIV-6 induced *unpaired* expression A)** S2* cells were infected with IIV-6 for the indicated times and induction of *upd1*, *upd2*, or *upd3* was monitored by qRT-PCR, compared to PBS injected controls. Three biologically independent replicates are shown and statistical analysis was performed by Multiple t-tests with the Holm-Sidak correction for multiple comparisons. Error bars indicate standard deviation and black bars indicate the mean. **B)** *TotA* or *TotM* expression was measured by qRT-PCR from control *w*^*1118*^ flies as well as *outstretched*^*s*^ (*os*^*s*^), *upd2*^*Δ*^, *upd3*^*Δ*^, *or upd2*^*Δ*^,*3*^*Δ*^ mutant flies 24 hours after IIV-6 infection or PBS injection. The results of 3–5 biologically independent assays are displayed. Error bars represent the standard deviation and black bars represent the mean. Statistical significance compared to the IIV-6 infected control strain (*w*^*1118*^) was determined by two-way ANOVA with Holm-Sidak correction. **C)** S2* cells were treated with inhibitors for the three MAPKs: JNK (SP600125, 25μΜ), ERK (U0125, 10μΜ), and p38 (SB203580, 10μΜ), or treated with a vehicle control (DMSO), for one hour prior to IIV-6 infection or mock treatment. After 24 hours of infection, *upd3* expression was assayed by qRT-PCR. The results of 3 biologically independent assays are shown. Statistical significance, compared to the DMSO treated control, was determined by two-way ANOVA with Holm-Sidak correction. **D)** S2* cells were treated with the JAK inhibitor Tofacitinib (CP690,550 10 μM), or treated with a vehicle control (DMSO), for one hour prior to IIV-6 infection or mock treatment. After 24 hours of infection, *TotA* and *upd3* expression were assayed by qRT-PCR. 3 biologically independent assays with statistical significance, comparing virus infected vehicle to inhibitor treatments, determined by two-way ANOVA with Holm-Sidak's multiple comparisons test. Significance is indicated in A-D as * *p* < 0.05; ** *p* < 0.01; *** *p* < 0.001; **** *p* < 0.0001; and ns, not significant. A.U., Arbitrary Units.

Next, we hypothesized that IIV-6 infection may induce the expression of one or more of the *unpaireds*, which encode the ligands for the gp130-like receptor Domeless. Unpaired 1, 2, and 3 are all distantly related to IL-6 [27]. *Unpaired 1* is critical for embryonic development, and *upd1* null flies are embryonic lethal, while the *upd2*, *upd3* double deletion is viable. *Unpaired 3* is induced in hemocytes after septic injury [26] and plays a role in gut regeneration following damage [28]. In S2* cells, IIV-6 infection induced expression of all three *unpaireds*, ~10^4^-fold as measured by qRT-PCR (Fig 4A). Hypomorphic alleles of *upd1*, also known as *outstretched*, are viable and some of these alleles, such as *os*^*s*^, also affect the expression of *upd3*, which lies nearby [29]. In adult flies, IIV-6 induced *TotA* expression was significantly reduced in all *upd* alleles, with the largest decrease in the *os*^*s*^ allele (Fig 4B). *TotM* induction was similarly reduced by *os*^*s*^ to levels observed in the PBS injected control, with lesser but still significant reductions in the *upd3* mutant and *upd2*^*Δ*^,*upd3*^*Δ*^ double mutant. Given the phenotypes in the hypomorphic *os*^*s*^ allele and the *upd2*^*Δ*^*upd3*^*Δ*^ double mutant, these data indicate that the *unpaireds* function redundantly to drive JAK-STAT signaling in response to IIV-6 infection.

The IIV-6 triggered expression of *upds* suggests that virus infection may induce these cytokine genes, which in turn will drive JAK-STAT signaling and *Tot* expression. In other systems, such as gut renewal, it has been suggested that various MAPK pathways are responsible for driving *upd* expression [28, 30–32]. Therefore S2* cells were treated with inhibitors targeting the three *Drosophila* MAPKs (JNK, p38, or ERK) and then infected with IIV-6. Treatment with p38 inhibitor significantly reduced *upd3* expression to near baseline levels, while ERK inhibitor had no effect and JNK inhibitor actually increased *upd3* expression (Fig 4C). On the other hand, the JAK-STAT inhibitor Tofacitinib blocked IIV-6 induced *TotA* expression but had no effect on the expression of *upd3* (Fig 4D). Together, these data indicate that while JAK-STAT is required for *Tot* induction, the virus-triggered expression of the *upds* (JAK-STAT activating cytokines) involves the p38 MAPK pathway.

### *In vivo*, p38b is required for IIV-6 induced *TotA* expression and survival from infection

**Fig 5:**
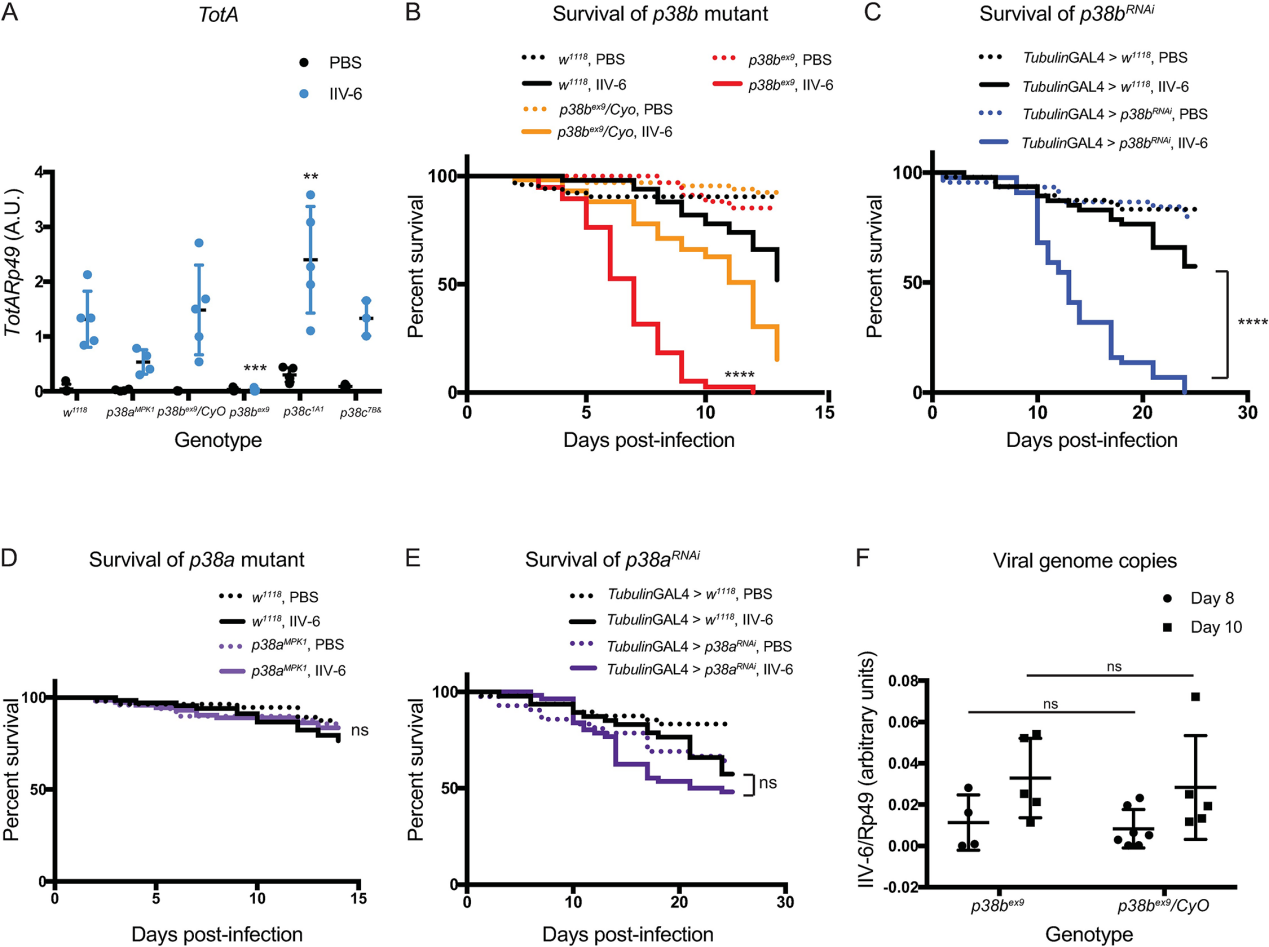
***p38b* is required for *turandot* induction and survival A)** Mutant strains for the three p38 homologs, *p38a*^*MPK1*^, *p38b*^*ex9*^, or *p38c*^*1A1*^, were infected with IIV-6 or injected with PBS, and *TotA* expression was measured by qRT-PCR 24 hours post-infection. Control genotypes included *w*^*1118*^ and *p38b*^*ex9*^/*CyO* heterozygotes. The results of 3–5 biologically independent replicates are shown. Black bars indicate the mean and error bars represents standard deviation. Statistical analysis, comparing *TotA* levels in the virus infected control to mutant strains, was determined by two-way ANOVA with Sidak’s correction for multiple comparisons (***p = 0.0004, ** p = 0.0045) **B)** Kaplan-Meier curves showing survival of homozygous (*p38b*^*ex9*^, red lines) or heterozygous (*p38b*^*ex9*^*/CyO*, orange lines) *p38b* mutant flies following IIV-6 infection (solid lines) or PBS-injected controls (dotted lines) compared to control (*w*^*1118*^, black lines) flies. **C)** Kaplan-Meier curves showing survival of *p38a* mutant flies (red lines) following IIV-6 infection (solid lines) or PBS-injected controls (dotted lines) compared to control (*w*^*1118*^, black lines) flies. **D)** Kaplan-Meier curves showing survival of *p38b*^*RNAi*^ expressing (blue lines) or **E)** *p38a*^*RNAi*^ expressing flies (purple lines) following IIV-6 infection (solid lines) or PBS-injected controls (dotted lines). *UAS-p38a*^*RNAi*^ and *UAS-p38b*^*RNAi*^ flies were crossed to *tubulin-GAL4* for ubiquitous knock-down, while the control was generated by *w*^*1118*^ crossed to *tubulin-GAL4* (black lines). Statistical significance was determined by Log-rank (Mantel-Cox) test (**** p <0.0001; ns, not significant). A.U., Arbitrary Units. **F)** Viral loads as determined by QPCR for *p38b*^*ex9*^ and *p38b*^*ex9*^*/CyO* heterozygous siblings. Each data point represents 5 flies. Error bars indicate standard error, and black lines indicate the mean. A.U., arbitrary units. Statistics were determined using two-way ANOVA. ns, not significant.

*Drosophila* encode three p38 homologs, *p38a*, *p38b*, and *p38c*, with *p38a* and *p38c* juxtaposed on third chromosome and p38b on the second. *p38a/c* mutant flies are susceptible to oxidative stress and heat shock, though not to osmotic shock [33], while *p38b* has been shown to provide protection against pathogenic bacterial or fungal challenge, although the underlying mechanisms are unclear [34]. *p38c* has also been implicated in gut homeostasis and reactive oxygen species (ROS) production in the gut upon infection with *P*. *entomophila* or *Erwinia carotovora carotovora 15* [35]. In addition, *p38c* is required for the induction of *DOPA decarboxylase*, which is required for the production of antimicrobial quinones produced in response to wounding [36]. *p38b* has also been linked to tolerance to infection with *Salmonella typhimurium* [37]. Mutant flies for all three p38 homologs are viable to adulthood and were infected with IIV-6 to determine their ability to induce *Tots*. We found that *p38a* and *p38c* null flies displayed normal levels of *Tot* expression following IIV-6 infection, while *p38b* null flies had only basal levels of *Tot* expression (Fig 5A). Furthermore, *p38b* heterozygous flies also expressed wild-type levels of *Tots* upon IIV-6 infection. Consistent with these *TotA* expression data, *p38b* mutants but not *p38a* mutants were hypersusceptible to IIV-6 infection (Fig 5B and 5C, with additional trials in S4 and S5 Figs). Likewise, *p38b* RNAi, when expressed with a ubiquitous driver (*tubulin-GAL4*), exhibited significantly reduced survival following IIV-6 infection compared to wild-type (driver alone) controls (Fig 5D, additional trials S6 Fig), while *p38a*^*RNAi*^ lines survived similarly to controls (Fig 5E, additional trials, S7 Fig). The knock-down efficiencies for these lines are shown in S3 Fig These combined results show that *p38b* is required for *Tot* induction and survival following IIV-6 infection in adult flies. The S2* cell data further demonstrates that p38 is required for *unpaired* induction. Together, these data suggest that some aspect of viral infection triggers p38b activation leading to Unpaired production, JAK-STAT activation, and *Tot* induction.

These results prompted us to ask whether these survival defects indicate a direct antiviral role for p38b, or whether p38b promotes tolerance to IIV-6. To discern between these possibilities, viral genomes were quantified by QPCR from *p38b* heterozygous and homozygous strains. *p38b* homozygous mutant flies showed no increase in viral titer compared to heterozygous *p38b* siblings, suggesting that p38b promotes tolerance (Fig 5F).

**Fig 6:**
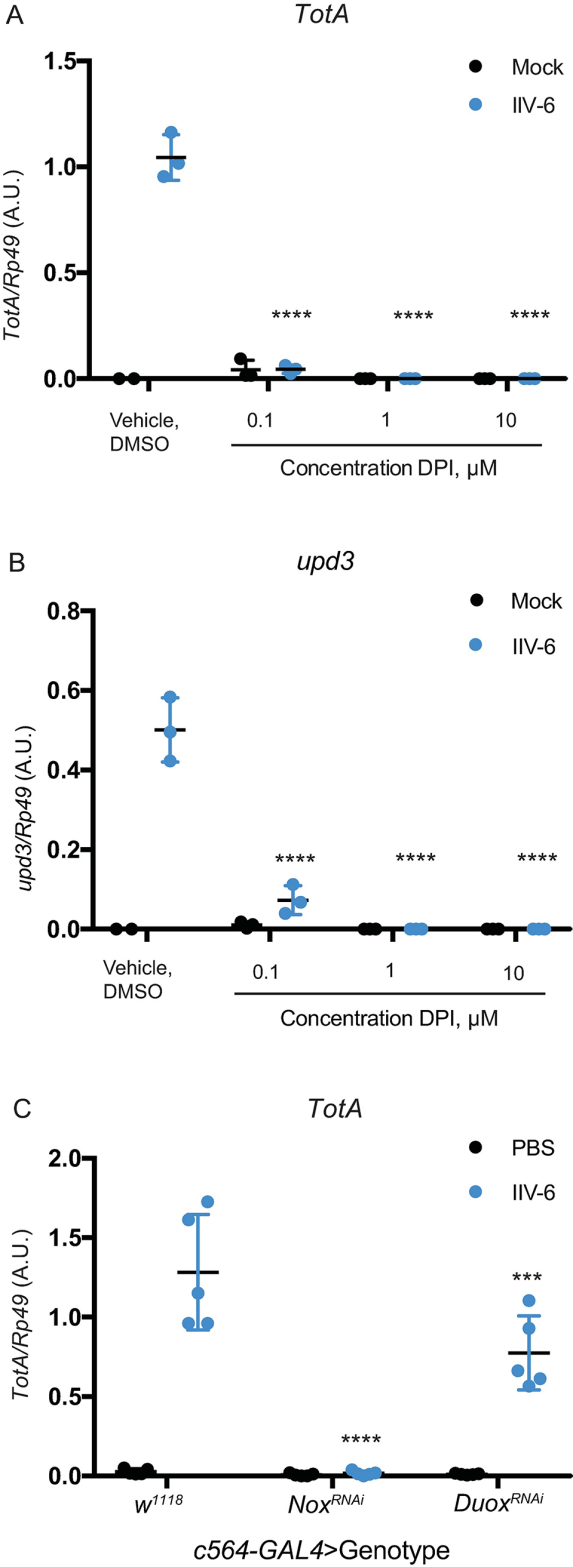
***Nox* is required for IIV-6 induced *upd3* and *turandot* expression** S2* cells were treated with the indicated concentrations of DPI (an NADPH oxidase inhibitor) or vehicle control (DMSO) for one hour, and then infected with IIV-6 or mock treated. **A)** *TotA* expression and **B)** *unpaired3* expression were quantified by qRT-PCR 24 hours post-infection. For **A)** and **B)**, 3 biologically independent replicates are shown, and statistics performed comparing virus infected vehicle treated to DPI treated conditions. **C)** *UAS-Nox*^*RNAi*^ and *UAS-Duox*^*RNAi*^ flies were crossed to *c564-GAL4* for fat body specific knock-down, while the control was generated by *w*^*1118*^ crossed to *c564-GAL4*. Progeny were infected with IIV-6 or injected with PBS, and *TotA* expression was measured in whole flies by qRT-PCR 24 hours post-infection. 5 biologically independent replicates are shown. For all panels, error bars represent standard deviation, black lines indicate the mean, and statistical significance was determined using two-way ANOVA with Sidak’s correction.(***p = 0.0004, ****p<0.0001). A.U., Arbitrary Units.

Viral infection damages cells, often inducing cell death (both apoptotic and necrotic) as well as the acute production of antivirals. These responses to viral infections can also trigger the release of endogenous activators of inflammation, so called DAMPs. These DAMPs often lead to MAPK activation. In particular, it has been shown that ROS can trigger p38 MAPK signaling [38][31]. Therefore, we sought to determine whether ROS generation is required for IIV-6-triggered, p38-dependent *unpaired* expression. To this end, we treated S2* cells with diphenyleneiodonium chloride (DPI), an NADPH oxidase inhibitor, prior to infection with IIV-6. DPI treatment completely abrogated induction of both *upd3* (Fig 6A) and *TotA* (Fig 6B) 24 hours after IIV-6 infection. To investigate which of the two NADPH oxidases encoded by *Drosophila* is responsible for this effect, we utilized previously characterized RNAi lines targeting each oxidase [39], with a fat-body specific expression (c564 driver). *Nox* knockdown, but not *Duox*, prevented IIV-6-triggered *TotA* induction (Fig 6C). It is noteworthy that *Nox* has previously been implicated to function in the sterile injury response, in particular the response to extracellular actin, within the fat body [38]. These results argue that IIV-6 infection triggers ROS production through Nox, that, in turn, stimulates p38b activation, Upd production and *Tot* induction.

## Discussion

**Fig 7:**
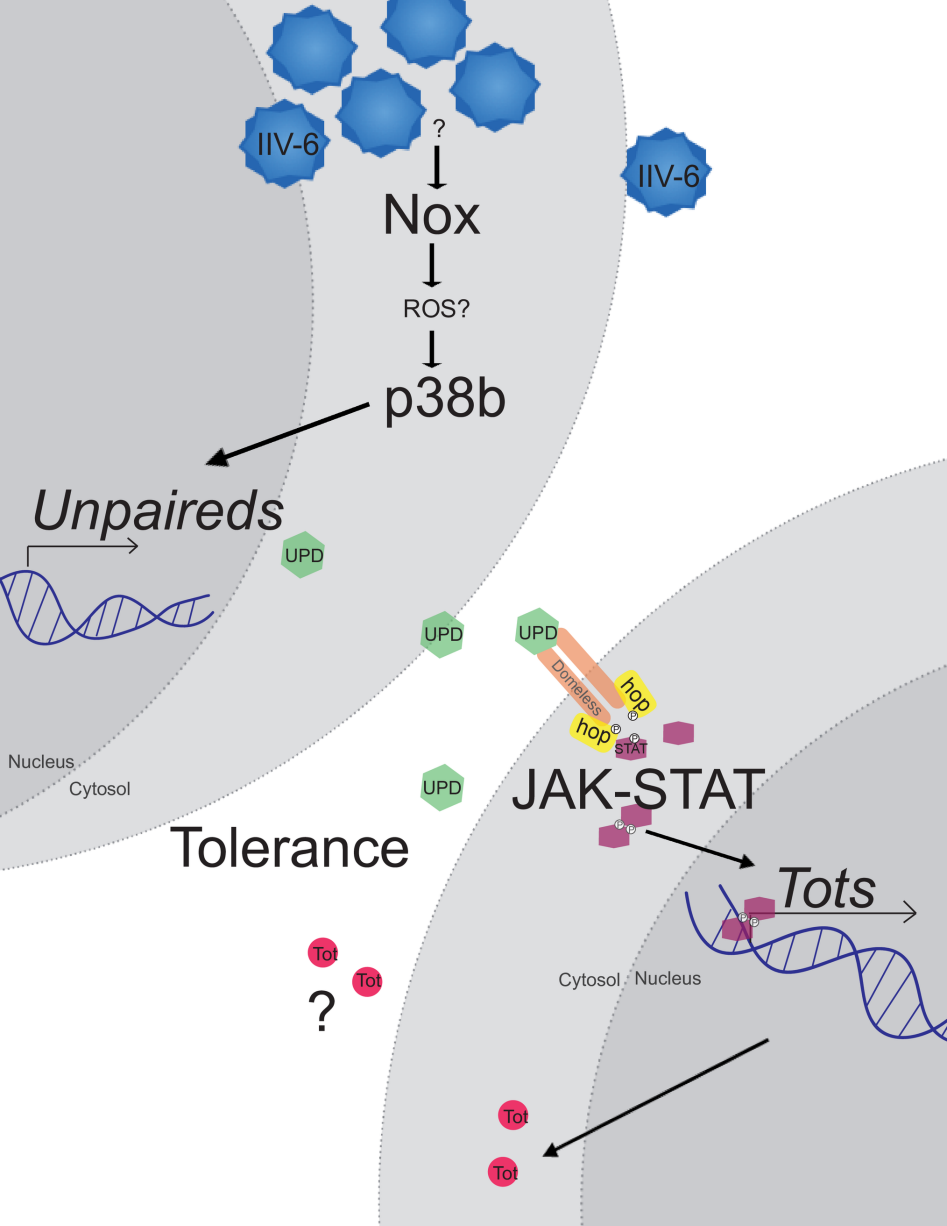
**IIV-6 infection activates a protective response through p38b and JAK-STAT signaling** IIV-6 infection activates p38b through the NADPH oxidase Nox and presumably ROS production. p38b triggers the induction of the *unpaireds*, which encode for secreted ligands which activate the JAK-STAT pathway via the receptor, Domeless. JAK-STAT signaling leads to induction of the *Turandots (Tots)*, a family of small, secreted proteins with no known function. JAK-STAT as well as p38b signaling protects *Drosophila* from IIV-6 infection, and p38b in particular increases tolerance to this virus.

Here, we show that infection of *Drosophila* with the DNA virus IIV-6 triggers a protective p38b-dependent response (see pathway model in Fig 7). While previous work has demonstrated that *Drosophila* p38b is critical for survival to bacterial or fungal infections and affects the tolerance to bacterial infections [34, 37], this is the first time p38b has been linked to antiviral defenses. Critical targets for p38b for the protection against IIV-6 infection are the *unpaireds*, a family of three IL-6-like genes clustered together on Chromosome X. The genetic data presented here argue that the three Unpaireds function together, in a partially redundant manner, to activate the JAK-STAT pathway following IIV-6 infection, thereby driving *Tot* gene expression. The JAK-STAT pathway also protects against IIV-6 infection, although the role of the Tots in antiviral defense requires more study. These results also imply that p38b is activated following IIV-6 infection. While the mechanisms leading from virus infection to p38 activation are unclear, they likely involve ROS-mediated signaling as the induction of *TotA* expression is potently blocked by an NADPH oxidase inhibitor and require the *Nox* gene. This is reminiscent of the activation of p38a by ROS generated from apoptotic cells in models of tissue regeneration [31].

Interestingly, p38b has also been shown to provide tolerance to *Salmonella typhimurium* infections, promoting survival of the host without reducing bacterial burden [37]. This study suggested that p38b contributes to tolerance by enabling hemocyte enlargement, and hence, engulfment of larger quantities of bacteria. In the context of IIV-6 infection, p38b could be acting to promote engulfment of infected and damaged cells, thereby providing a repair mechanism to enable the animals to better tolerate and limit virus infection. Future studies will be necessary to probe all the roles of p38b in antiviral defense.

Although the data presented here demonstrate that the JAK-STAT pathway is protective against IIV-6 infection, the protective mechanisms require further study. In the case of the RNA virus DCV, the JAK-STAT pathway is also protective, possibly through the induction of *vir-1*. However, the JAK-STAT pathway is not broadly antiviral and *vir-1* was not induced by IIV-6 [8]. Curiously, a previous study examining the role of the JAK-STAT pathway during IIV-6 infection, using one particular hypomorphic allelic combination *hopscotch* (JAK), concluded that hopscotch (and by inference the JAK-STAT pathway) was not involved in protecting flies against IIV-6 infection [7]. Our data, with multiple RNAi lines targeting *stat92E*, as well as the S2 cell based results with RNAi targeting *domeless*, *hopscotch*, and *stat92E*, demonstrate a consistent and reproducible role for this pathway in the response to and survival from IIV-6 infection. We believe these contradictory outcomes may be due to differences in alleles used or dose delivered.

The Tots are intriguing candidates for JAK-STAT induced antivirals. They are rapidly evolving with evidence of positive selection, typical for immune effectors [20, 40]. However, the Tots have not yet been demonstrated to provide direct antimicrobial activity. To date, we have been unable to demonstrate any antiviral activity for the Tots. In particular, over-expression of *TotA* resulted in reduced survival following IIV-6 infection and no change in viral titers (S8 Fig), consistent with the previously reported general toxicity caused by over expression of this gene [21]. Further studies, examining all six of the IIV-6 induced *Tots*, with both loss- and gain-of-function approaches, will be necessary to more fully examine this possibility.

The sensitivity of STAT knockdowns to IIV-6 infection argues that JAK-STAT signaling is an important antiviral target of p38b. However, other p38b targets are also possible. For example, an established target of p38b is the heat shock response. In the context of bacterial and fungal infections, p38b is known to regulate Heat shock factor (Hsf) expression and the induction of heat shock proteins (Hsps) [34]. In addition, another report has shown that Hsf protects flies against both RNA and DNA viral infections [41]. Together, these results suggest that the antiviral effects of p38b could be mediated, at least in part, through Hsf and Hsps. Indeed, *Hsf* mutant flies display an increased rate of death after IIV-6 infection. It will be interesting to learn if the heat shock response is activated by p38b following IIV-6 infection, and how this response interacts with JAK-STAT dependent viral protection.

Successful host defenses detect multiple characteristics of an invading pathogen. For example, cellular damage is one common indicator of pathogenic infection that can be sensed by the innate immune system. In mammals, several danger-associated molecular patterns (DAMPs) have been characterized, including HMGB1, F-actin, and histones [42–44]. Likewise, a recent report examining a *Drosophila* model of sterile injury demonstrated that extracellular actin activates JAK-STAT signaling [38]. In this paradigm, detection of extracellular actin, via an unknown receptor, triggered Nox-dependent ROS generation, the activation of Src42A and Shark (Syk homolog), and induction of *unpaireds* and eventually *Tots*. This pathway is very similar to that reported here, although p38b was not examined in this actin-DAMP, and suggests that IIV-6 infection may cause cellular damage, rupture and the release of actin, which in turn triggers ROS production, *unpaired* expression, JAK-STAT signaling and the induction of *Tots*. Formally testing this model will be facilitated by the identification of an extracellular actin receptor.

In summary, we have found a novel role for *Drosophila* p38b in protecting against DNA virus infection. Virus infection leads to p38b dependent responses, including the induction of the JAK-STAT activating cytokines, the Unpaireds, and the induction of downstream target genes such as the *Tots*. Based on the analysis of viral load, the p38b pathway appears to function primarily by increasing tolerance to IIV-6, as viral loads were not altered in the *p38b* strain. Whether the Tots contribute to this tolerance and, more generally, whether p38b induces a directly antiviral response, or relies entirely on the Unpaired and JAK-STAT signaling for its ability to tolerize against this viral infection will be probed in future studies.

## Methods

### Reagents

p38 inhibitor SB203580 (CAT#13067) [45], JNK inhibitor SP600125 (CAT#10010466) [46], JAK inhibitor Tofacitinib CP690,550(CAT#11598) [47], and ERK inhibitor U-0126(CAT#70970) [48–50], were purchased from Cayman Chemical. Diphenyleneiodonium chloride DPI (CAS#4673-26-1) [39] was purchased from Sigma-Aldrich. All inhibitors were dissolved in DMSO and used at the indicated concentrations.

### Fly stocks and infections

*p38a*^*MPK1*^, *p38b*^*ex9*^*/CyO*, *p38c*^*1A1*^*/TM6*, and *p38c*^*7B&*^*/TM6* flies were a kind gift of Bruno Lemaitre. *UAS-TotA* flies were a kind gift of Dan Hultmark. *Nox*^*RNAi*^ and *Duox*^*RNAi*^ lines were generated by Won-Jae Lee [39] and obtained from Andreas Bergmann. stat92E^RNAi^, *w*^*1118*^*; P{UAS-STAT92E*^*GD4492*^*RNAi}v43866*, and *w*^*1118*^*; P{UAS-p38a*^*GD17018*^*RNAi}v52277* were obtained from the Vienna Drosophila Resource Center (VDRC). *y*^*1*^ *v*^*1*^*; P{p38b*^*TRiP*.*JF03341*^*RNAi}attP2*, *Ab(1)os*^*s*^, *upd1*^*os-s*^ *upd3*^*os-s*^(BIN#79), *Df(1)os*^*o*^, *upd1*^*os-o*^ *upd3*^*os-o*^(BIN#78),w^*^ *upd2*^*Δ*^ (BIN#55727), w^*^ *upd3*^*Δ*^ (BIN#55728),w^*^ *upd2*^*Δ*^*upd3*^*Δ*:^ (BIN#55729), stat92E^RNAi^-2, y^1^ v^1^; P{TRiP.HMS00035} attP (BIN#33637), stat92E^RNAi^-1, y^1^ v^1^; P{TRiP.JF01265} attP2 (BIN#31317), were obtained from Bloomington Drosophila Stock Center (BDSC). *w*^*1118*^ flies were used as an immunologically wild-type control in all experiments, as these are the most similar background to the alleles listed above.

Three to five day old flies, maintained at 22°C, were used for all experiments. Flies were injected intrathoracically with 32.2 nL of virus (1x10^4^ TCID50) or vehicle (PBS) using a Nanoject II (Drummond). For survival assays, a minimum of fifty flies were used per treatment, per genotype and the dead were counted daily. Kaplan-Meier curves are shown and significance was determined by log-rank (Mantel-Cox) using GraphPad Prism. For qRT-PCR analysis, at least three independent replicates of 15–20 flies each were used for RNA extraction. In all cases, three or more independent replicates, as indicated in each figure legend, were performed in parallel on the same day. At least 2 additional trials, each with 3 or more biologically independent replicates, were performed at other times, with similar results.

### nCounter analysis

The expression levels of 139 Drosophila immune genes were assayed from 100 nanograms of RNA via a customized Nanostring nCounter codeset. Two biological replicates of 10–15 adult male flies 3–5 days of age were analyzed for each treatment and timepoint. The results were analyzed using nSolver 3.0 software according to the manufacturers instructions (NanoString Technologies, Seattle, WA, USA), and the heatmap was created using nSolver 3.0 software and JavaTree.

### RNA isolation and qRT-PCR

Total RNA from flies or S2* cells was extracted using TRIzol (Invitrogen). Samples were then DNase treated (RQ1, Promega) and RNA re-extracted by phenol-chloroform. cDNA was synthesized using iScript cDNA Synthesis kit (BioRad). Alternatively, the gDNAclear cDNA synthesis kit (BioRad) was used following TRIzol purification. qRT-PCR was analyzed normalizing to the housekeeping gene Rp49. Primer sequences can be found as S1 Table. Cycling conditions: 50°C, 2 minutes; 94°C, 2 minutes; 95°C, 15 seconds; 61°C, 30 seconds; 72°C, 30 seconds; plate read, amplification cycle repeated 39 times. Melt curve performed and plate read, 58°C-95°C, 0.5°C increments. 10°C for 5 minutes. Cycling conditions for IIV-6 QCPR: 50°C, 2 minutes; 94°C, 2 minutes; 95°C, 15 seconds; 72°C, 1 minute; plate read, two-step amplification cycle repeated 39 times. Melt curve performed and plate read, 58°C-95°C, 0.5°C increments. 10°C for 5 minutes.

### Cell culture and RNAi

dsRNA was produced as previously described, using primers and cycling conditions as recommended by the DRSC (Drosophila RNAi Screening Center) [51, 52]. Primer sequences can be found in S1 Table. S2* cells were cultured as previously described [53, 54] and were transfected with 3μg of dsRNA using Cellfectin II reagent (Invitrogen). Cells were split 24 hours after transfection to 5x10^5^ cells/mL and 24 hours later cells were infected with IIV-6 at an MOI of 2. As a control, cells were mock-treated with the same volume of PBS (virus diluent) as used in infections. Cells were harvested in TRIzol (Invitrogen) 24 hours post-infection. In experiments with small molecule inhibitors, cells were treated with the indicated inhibitor at the stated concentration or the appropriate vehicle control 1 hour prior to virus infection.

### Virus preparation

IIV-6 was provided by Luis Teixeira. IIV-6 was propagated and purified on DL-1 cells as previously described (9), with a final resuspension in PBS, and quantified on DL-1 cells by TCID50. Cells were infected at an MOI of 2 unless otherwise noted, while flies were injected with 1x10^4^ TCID50, as detailed above.

## Supporting information

S1 Table Primer sequences used for RT-PCR, QPCR, or dsRNA synthesis. (PDF)

S2 Table Hazard ratios for IIV-6-infected or PBS-injected flies. In each case the mutant or RNAi line of interest (Genotype A) is compared to the control line (Genotype B). p value is indicated for each comparison, and the Figure and Panel is indicated at right.
(PDF)

S1 Fig **A-C)** RT-PCR showing knockdown efficiencies of **A)** *stat92E*, **B)** *domeless*, and **C)** *hopscotch* in S2* cells. Expression levels were normalized to *Rp49*, and are shown as % Max Expression, determined by expression of control samples Raw p values are shown above relevant data. **D)** RT-PCR showing knockdown efficiencies of *stat92E*^*RNA*i^ fly lines crossed to c564-GAL4. *stat92E*^*RNAi*^ VDRC; *stat92E*^*RNAi*^*-1*, P{TRiP.JF01265}; *stat92E*^*RNAi*^*-2*, P{TRiP.HMS00035}. Expression levels were normalized to *Rp49*, and are shown as % Max Expression, with maximum determined by expression of control *w*^*1118*^ samples. **D)** Statistics were determined by one-way ANOVA. *p<0.05, **p<0.01, ***p<0.001. **E)** Kaplan Meier curve showing survival of *stat92E*^*RNAi*^*-2*, under control of *c564-GAL4* (green lines), following IIV-6 infection (solid lines) or PBS-injection (dotted lines). *w*^*1118*^ (black lines) are used as control flies. Statistical significance was determined by Log-rank (Mantel-Cox) test, comparing IIV-6 infected RNAi lines to IIV-6 infected control animals, or comparing PBS-injected RNAi lines to PBS-injected control lines. **p <0.005. ns, not significant.
(PDF)

S2 Fig Replicate trials of *stat92E*^*RNAi*^ (VDRC) survival curves. Kaplan-Meier curves showing survival of *Stat92E*^*RNAi*^ expressing (*UAS-Stat92E*^*RNAi*^ *x tubulin-Gal4)* flies (green lines) or control (*w*^*1118*^ *x tubulin-Gal4)* flies (black lines) following infection with IIV-6 (solid lines) or injection with PBS (dotted lines). Results shown are for 50 flies per genotype and treatment. Statistical significance was determined by Log-rank (Mantel-Cox) test, comparing IIV-6 infected RNAi lines to IIV-6 infected control animals, or comparing PBS-injected RNAi lines to PBS-injected control lines. ****p <0.0001. ns, not significant.
(PDF)

S3 Fig RT-PCR showing knockdown efficiencies for progeny of **A)** *p38b*^RNAi^ fly lines crossed to *Tubulin-GAL4* or **B)** *p38a*^RNAi^ fly lines crossed to *Tubulin-GAL4*. Expression levels were normalized to *Rp49*, and are shown as % Max Expression, with maximum determined by expression of control (*w*^*1118* x^*Tubulin-GAL4*) flies. Statistical significance was determined by two-tailed unpaired t test, *p<0.05, **p<0.005.
(PDF)

S4 Fig Replicate trials of *p38b*^*ex9*^ survival curves. Kaplan-Meier curves showing survival of homozygous (*p38b*^*ex9*^, red lines) or heterozygous (*p38b*^*ex9*^*/CyO*, orange lines) *p38b* mutant flies following IIV-6 infection (solid lines) or PBS-injected controls (dotted lines) compared to control (*w*^*1118*^, black) flies. Statistical significance was determined by Log-rank (Mantel-Cox) test, comparing IIV-6 infected mutants to IIV-6 infected control animals, or comparing PBS-injected mutants to PBS-injected control animals. *p<0.05, ****p <0.0001. ns, not significant.
(PDF)

S5 Fig Replicate trials of *p38a*^*MPK1*^ survival curves. Kaplan-Meier curves showing survival of *p38a* mutant flies (red lines) following IIV-6 infection (solid lines) or PBS-injected controls (dotted lines) compared to control (*w*^*1118*^, black) flies. Statistical significance was determined by Log-rank (Mantel-Cox) test, comparing IIV-6 infected mutants to IIV-6 infected control animals, or comparing PBS-injected mutants to PBS-injected control animals. *p<0.05, ****p <0.0001. ns, not significant.
(PDF)

S6 Fig Replicate trials of p38bRNAi survival curves. Kaplan-Meier curves showing survival of p38bRNAi expressing flies following IIV-6 infection (solid lines) or PBS-injected controls (dotted lines). *UAS-p38b*^*RNAi*^ flies were crossed to *tubulin-GAL4* for ubiquitous knock-down (blue lines), while the control was generated by *w*^*1118*^ crossed to *tubulin-GAL4* (black lines). Statistical significance was determined by Log-rank (Mantel-Cox) test, comparing IIV-6 infected RNAi lines to IIV-6 infected control animals, or comparing PBS-injected RNAi lines to PBS-injected control lines.**** p <0.0001; ns, not significant.
(PDF)

S7 Fig Replicate trials of *p38a*^*RNAi*^ survival curves. Kaplan-Meier curves showing survival of *p38a*^*RNAi*^ expressing flies following IIV-6 infection (solid lines) or PBS-injected controls (dotted lines). *UAS-p38a*^*RNAi*^ flies were crossed to *tubulin-GAL4* for ubiquitous knock-down (purple lines), while the control was generated by *w*^*1118*^ crossed to *tubulin-GAL4* (black lines). Statistical significance was determined by Log-rank (Mantel-Cox) test, comparing IIV-6 infected RNAi lines to IIV-6 infected control animals, or comparing PBS-injected RNAi lines to PBS-injected control lines.**** p <0.0001; ns, not significant.
(PDF)

S8 Fig A) Kaplan-Meier curve showing survival of *TotA* over-expressing flies or control (*w*^*1118*^) flies under the control of a fat body (*c564-GAL4*) or ubiquitous (*Tubulin-GAL4*) driver, infected with IIV-6. ****p<0.0001. n>50. **B)** Viral loads from TotA over-expressing flies or control (*w*^*1118*^) flies under the control of a fat body (*c564-GAL4*) or ubiquitous (*Tubulin-GAL4*) driver infected with IIV-6 and assayed by limiting dilution (TCID50) post-infection. TCID50 was calculated using Reed-Muench method. Each data point represents 5 flies. ns, no significance.
(PDF)
