# Supplementary material for: p38b and JAK-STAT signaling protect against Invertebrate iridescent virus 6 infection in Drosophila
Source: PLoS Pathog. 2018 May 10;14(5):e1007020. doi: 10.1371/journal.ppat.1007020 (PMC5963806; doi:10.1371/journal.ppat.1007020)
Supplement: S1 Table — (PDF) [file ppat.1007020.s001.pdf]

**S1 Table: Primer Sequences Used for RT-PCR, QPCR, or dsRNA synthesis**

| Primer | Sequence |
|---|---|
| TotA F | CCCAGTTTGACCCCTGAG |
| TotA R | GCCCTTCACACCTGGAGA |
| TotM F | ACCGGAACATCGACAGCCTAGCCA |
| TotM R | CCAGAATCCGCCTTGTGC |
| TotB F | CTGATTGGAACTCTATGCTCGGC |
| TotB R | TTGTCGTCCCAGGAGGTTCG |
| TotC F | TACTATGCCTTGCCCTGCTCC |
| TotC R | TGTTCAGGGGACAACGTGGG |
| TotE F | GGCTGCCTTTTGGGTTCAGG |
| TotE R | TATCGACGGAGGGATTGCCG |
| TotF F | AAGGCACGTCAAATGCTCGC |
| TotF R | TGTTGGTTGTTGTGTGCCCG |
| TotX F | TCCATCATCCCATCAAGCAAGC |
| TotX R | GCACCATCAACCAAAACTGCC |
| TotZ F | CTCACAGGAAACGGAAGTGC |
| TotZ R | GCGGCCTTCTTTATTGCTCC |
| upd1 F | CGATCATCCGACCCGTAATCC |
| upd1 R | ACTGTTCCTCCCTGACTAGATGG |
| upd2 F | TCAACTTTCACACGCCAGCC |
| upd2 R | CACCAGTTCCGTGAATCGCC |
| upd3 F | ACAAAACGGCCAGAACCAGG |
| upd3 R | GAAGGTCAGCCGGAAGTTGG |
| IIV-6 MCP F | GTATGGCAAAGCAGCAGTAGGAAGAGCAACTCCAGAATCGC |
| IIV-6 MCP R | TCTCGCGATCGTAACTATTCTCTAATTTTTTTACTTGCATGAAAAATAAG |
| hop T7-DRSC23334 F | TAATACGACTCACTATAGGGGACCACTCACCGACAATCCT |
| hop T7-DRSC23334 R | TAATACGACTCACTATAGGGCAGTCCCGTTGATTTGTCCT |
| hop T7 DRSC26551 F | TAATACGACTCACTATAGGGGAGTCCCGTCAAGGATGTGT |

**S1 Table: Primer Sequences Used for RT-PCR, QPCR, or dsRNA synthesis**

| | |
|---|---|
| hop T7 DRSC26551 R | TAATACGACTCACTATAGGGATAGCCGGGATCGCTAATTT |
| Domeless, T7 DRSC19583 F | TAATACGACTCACTATAGGGCGTCTGCGCAGTGATCC |
| Domeless, T7 DRSC19583 R | TAATACGACTCACTATAGGGTGGGCTCCGATGGATAGA |
| Domeless, T7 DRSC36980 F | TAATACGACTCACTATAGGGCGGGCAGGAACCTATCCTAT |
| Domeless, T7 DRSC36980 R | TAATACGACTCACTATAGGGGATATCGGACATCTTGCGGT |
| STAT92E, T7 DRSC16870 F | TAATACGACTCACTATAGGGCTTGCCCAAAACTACAGTTAC |
| STAT92E, T7 DRSC16870 R | TAATACGACTCACTATAGGGCGACTGTGGGTGGATTGTT |
| STAT92E, T7 DRSC37655 F | TAATACGACTCACTATAGGGGTGGCCCTAAAGTTCAACGA |
| STAT92E, T7 DRSC37655 R | TAATACGACTCACTATAGGGTGTTGCACCTCGTACAGCTC |
| domeless F | CGGCTACAACTACACGCTCC |
| domeless R | CCCAGTAGAAGCGCATAGCC |
| STAT92E F | AGTTCTACTCAAAGCGTCAAGATCC |
| STAT92E R | CAGTTGCATGCTTTCCTGAGC |
| hop F | AATTCGCTGAGTGTGGAGCG |
| hop R | GAGCGTGGACAGAGTGTTGG |
| p38a F | CAGCCCGTGGGATCGGG |
| p38a R | AGCTCCCGGTACGTCCTC |
| p38b F | CGGCCAGGTCTGCAAGGC |
| p38b R | CCATGTACACTTGCTGGAACTG |
| Rp49 F | CCCAAGGGTATCGACAACAG |
| Rp49 R | GACAATCTCCTTGCGCTTCT |
