# Supplementary material for: p38b and JAK-STAT signaling protect against Invertebrate iridescent virus 6 infection in Drosophila
Source: PLoS Pathog. 2018 May 10;14(5):e1007020. doi: 10.1371/journal.ppat.1007020 (PMC5963806; doi:10.1371/journal.ppat.1007020)
Supplement: S2 Table — In each case the mutant or RNAi line of interest (Genotype A) is compared to the control line (Genotype B). p value is indicated for each comparison, and the Figure and Panel is indicated at right. (PDF) [file ppat.1007020.s002.pdf]

S2 Table: Hazard Ratios of IIV-6-infected and PBS-injected flies.

Genotype A	Genotype B	Treatment	Hazard Ratio (logrank) A:B	p value Log Rank (Mantel Cox)	Figure/Panel
TubulinGAL4 > Stat92E^{RNAi} VDRC	*TubulinGAL4 > w^{1118}*	PBS	1.693	0.2752	Figure 3B
TubulinGAL4 > Stat92E^{RNAi} VDRC	*TubulinGAL4 > w^{1118}*	IIV-6	2.614	<0.0001	Figure 3B
c564GAL4 > Stat92E^{RNAi} (TRiP-1)	*w^{1118}*	PBS	1.389	0.2456	Figure 3C
c564GAL4 > Stat92E^{RNAi} (TRiP-1)	*w^{1118}*	IIV-6	2.708	<0.0001	Figure 3C
c564GAL4 > Stat92E^{RNAi} (TRiP-2)	*w^{1118}*	PBS	1.228	0.5451	Supplemental 1E
c564GAL4 > Stat92E^{RNAi} (TRiP-2)	*w^{1118}*	IIV-6	2.097	0.0066	Supplemental 1E
TubulinGAL4 > Stat92E^{RNAi} VDRC	*TubulinGAL4 > w^{1118}*	PBS	0.4437	0.0024	Supplemental Figure 2, Top
TubulinGAL4 > Stat92E^{RNAi} VDRC	*TubulinGAL4 > w^{1118}*	IIV-6	2.254	<0.0001	Supplemental Figure 2, Top
TubulinGAL4 > Stat92E^{RNAi} VDRC	*TubulinGAL4 > w^{1118}*	PBS	1.113	0.8238	Supplemental Figure 2, Bottom
TubulinGAL4 > Stat92E^{RNAi} VDRC	*TubulinGAL4 > w^{1118}*	IIV-6	4.123	<0.0001	Supplemental Figure 2, Bottom
p38b^{ex9}	*w^{1118}*	PBS	1.468	0.5402	Figure 5, B
p38b^{ex9}	*w^{1118}*	IIV-6	5.711	<0.0001	Figure 5, B
p38a^{MPK1}	*w^{1118}*	PBS	1.174	0.7441	Figure 5, C
p38a^{MPK1}	*w^{1118}*	IIV-6	0.6986	0.3922	Figure 5, C
TubulinGAL4 > p38b^{RNAi}	*TubulinGAL4 > w^{1118}*	PBS	1.189	0.7452	Figure 5D
TubulinGAL4 > p38b^{RNAi}	*TubulinGAL4 > w^{1118}*	IIV-6	4.643	<0.0001	Figure 5D

S2 Table: Hazard Ratios of IIV-6-infected and PBS-injected flies.

TubulinGAL4 > p38a^{RNAi}	*TubulinGAL4 > w^{1118}*	PBS	2.326	0.0445	Figure 5E
TubulinGAL4 > p38a^{RNAi}	*TubulinGAL4 > w^{1118}*	IIV-6	1.413	0.2149	Figure 5E
p38b^{ex9}	*w^{1118}*	PBS	3.738	0.0009	Supplemental Figure 4, Top
p38b^{ex9}	*w^{1118}*	IIV-6	8.09	<0.0001	Supplemental Figure 4, Top
p38b^{ex9}	*w^{1118}*	PBS	3.843	0.2068	Supplemental Figure 4, Bottom
p38b^{ex9}	*w^{1118}*	IIV-6	9.573	<0.0001	Supplemental Figure 4, Bottom
p38a^{MPK1}	*w^{1118}*	PBS	1.337	0.6062	Supplemental Figure 5, Top
p38a^{MPK1}	*w^{1118}*	IIV-6	0.1465	<0.0001	Supplemental Figure 5, Top
p38a^{MPK1}	*w^{1118}*	PBS	1.376	0.3507	Supplemental Figure 5, Bottom
p38a^{MPK1}	*w^{1118}*	IIV-6	0.5268	0.0104	Supplemental Figure 5, Bottom
TubulinGAL4 > p38b^{RNAi}	*TubulinGAL4 > w^{1118}*	PBS	0.5444	0.0703	Supplemental Figure 6, Top
TubulinGAL4 > p38b^{RNAi}	*TubulinGAL4 > w^{1118}*	IIV-6	3.723	<0.0001	Supplemental Figure 6, Top
TubulinGAL4 > p38b^{RNAi}	*TubulinGAL4 > w^{1118}*	PBS	1.268	0.6345	Supplemental Figure 6, Bottom
TubulinGAL4 > p38b^{RNAi}	*TubulinGAL4 > w^{1118}*	IIV-6	2.77	<0.0001	Supplemental Figure 6, Bottom
TubulinGAL4 > p38a^{RNAi}	*TubulinGAL4 > w^{1118}*	PBS	0.4507	0.0044	Supplemental Figure 7, Top
TubulinGAL4 > p38a^{RNAi}	*TubulinGAL4 > w^{1118}*	IIV-6	0.7262	0.2994	Supplemental Figure 7, Top
TubulinGAL4 > p38a^{RNAi}	*TubulinGAL4 > w^{1118}*	PBS	2.194	0.0866	Supplemental Figure 7, Bottom
TubulinGAL4 > p38a^{RNAi}	*TubulinGAL4 > w^{1118}*	IIV-6	0.6175	0.1433	Supplemental Figure 7, Bottom
