# Supplementary figures and images for: p38b and JAK-STAT signaling protect against Invertebrate iridescent virus 6 infection in Drosophila
Source: PLoS Pathog. 2018 May 10;14(5):e1007020. doi: 10.1371/journal.ppat.1007020 (PMC5963806; doi:10.1371/journal.ppat.1007020)

S1 Fig.

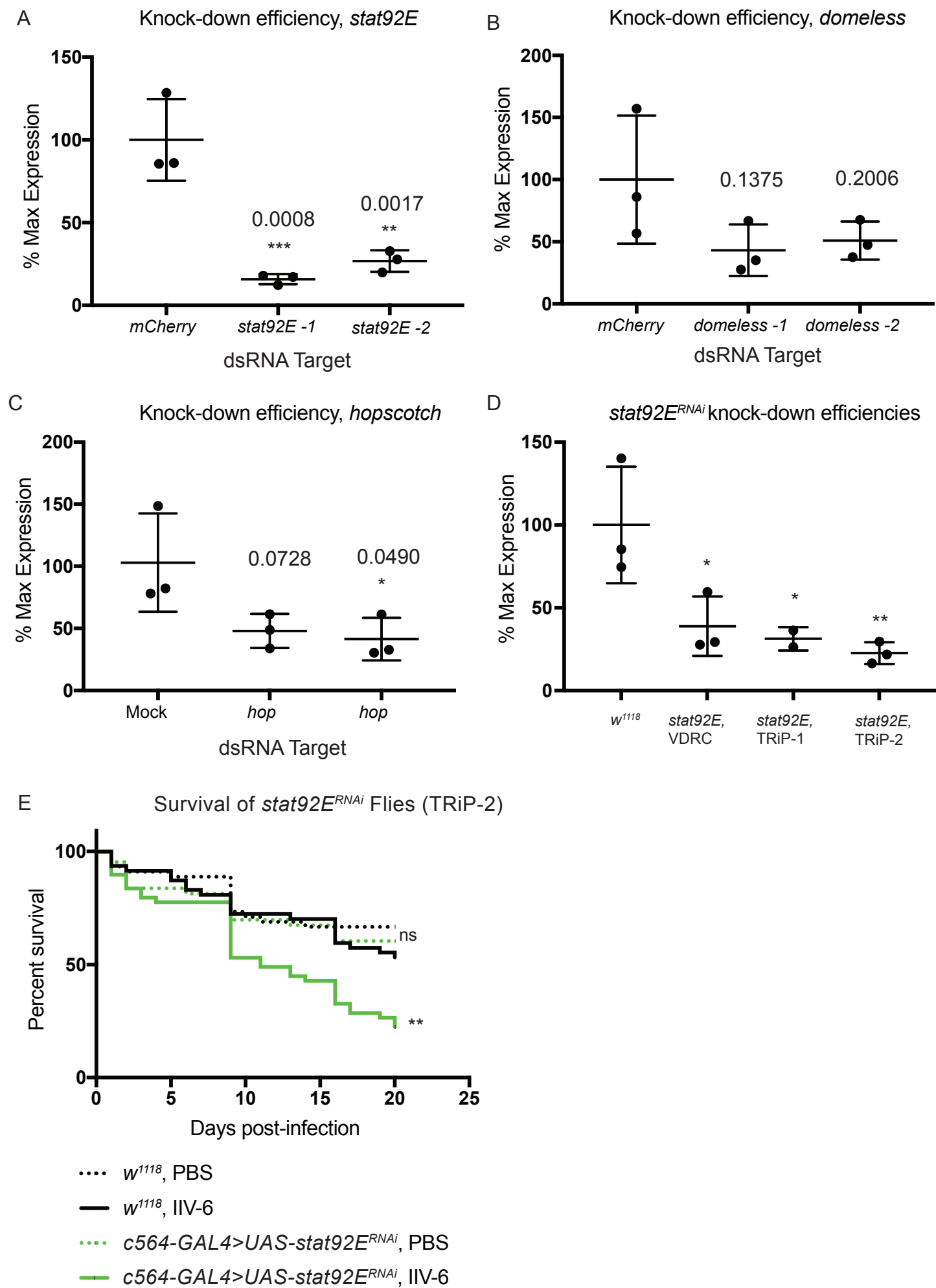

Supplement: S1 Fig — A-C) RT-PCR showing knockdown efficiencies of A) stat92E, B) domeless, and C) hopscotch in S2* cells. Expression levels were normalized to Rp49, and are shown as % Max Expression, determined by expression of control samples Raw p values are shown above relevant data. D) RT-PCR showing knockdown efficiencies of stat92ERNAi fly lines crossed to c564-GAL4. stat92ERNAi VDRC; stat92ERNAi-1, P{TRiP.JF01265}; stat92ERNAi-2, P{TRiP.HMS00035}. Expression levels were normalized to Rp49, and are shown as % Max Expression, with maximum determined by expression of control w1118 samples. D) Statistics were determined by one-way ANOVA. *p<0.05, **p<0.01, ***p<0.001. E) Kaplan Meier curve showing survival of stat92ERNAi-2, under control of c564-GAL4 (green lines), following IIV-6 infection (solid lines) or PBS-injection (dotted lines). w1118 (black lines) are used as control flies. Statistical significance was determined by Log-rank (Mantel-Cox) test, comparing IIV-6 infected RNAi lines to IIV-6 infected control animals, or comparing PBS-injected RNAi lines to PBS-injected control lines. **p <0.005. ns, not significant. (PDF) [file ppat.1007020.s003.pdf]

Survival of *stat92E<sup>RNAi</sup>* Flies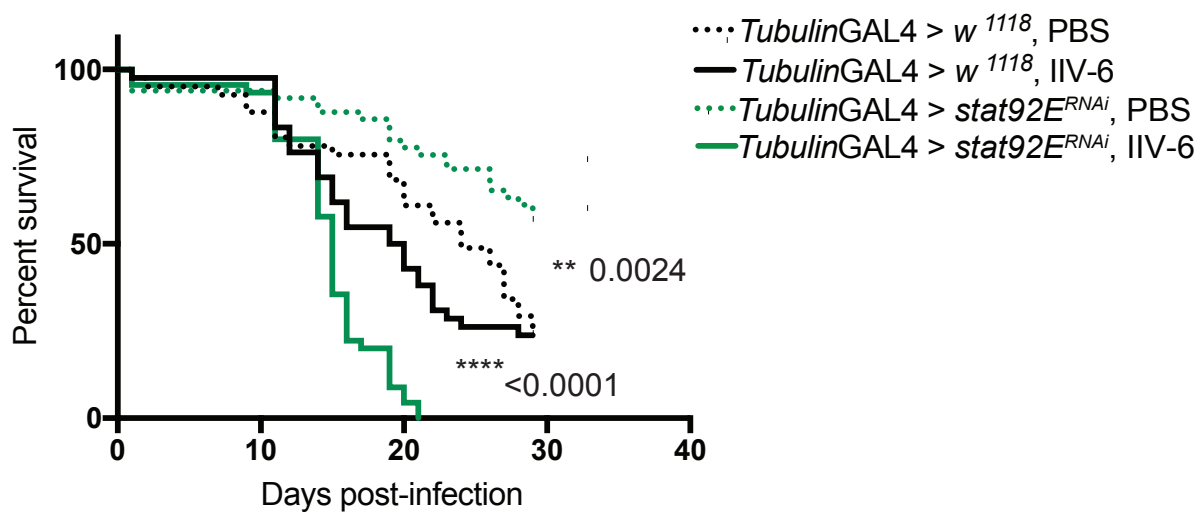Survival of *stat92E<sup>RNAi</sup>* Flies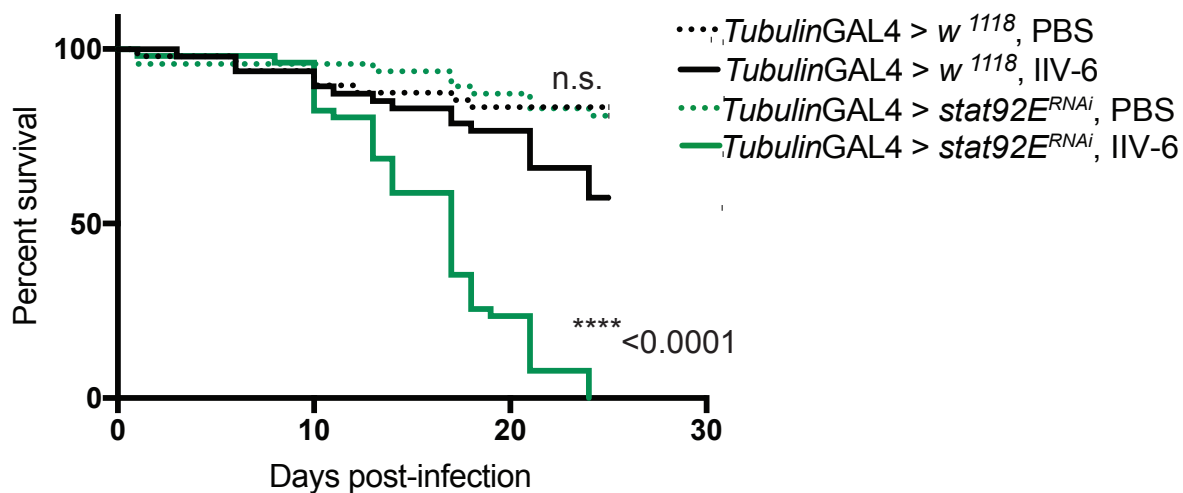

Supplement: S2 Fig — Kaplan-Meier curves showing survival of Stat92ERNAi expressing (UAS-Stat92ERNAi x tubulin-Gal4) flies (green lines) or control (w1118 x tubulin-Gal4) flies (black lines) following infection with IIV-6 (solid lines) or injection with PBS (dotted lines). Results shown are for 50 flies per genotype and treatment. Statistical significance was determined by Log-rank (Mantel-Cox) test, comparing IIV-6 infected RNAi lines to IIV-6 infected control animals, or comparing PBS-injected RNAi lines to PBS-injected control lines. ****p <0.0001. ns, not significant. (PDF) [file ppat.1007020.s004.pdf]

S3 Fig.

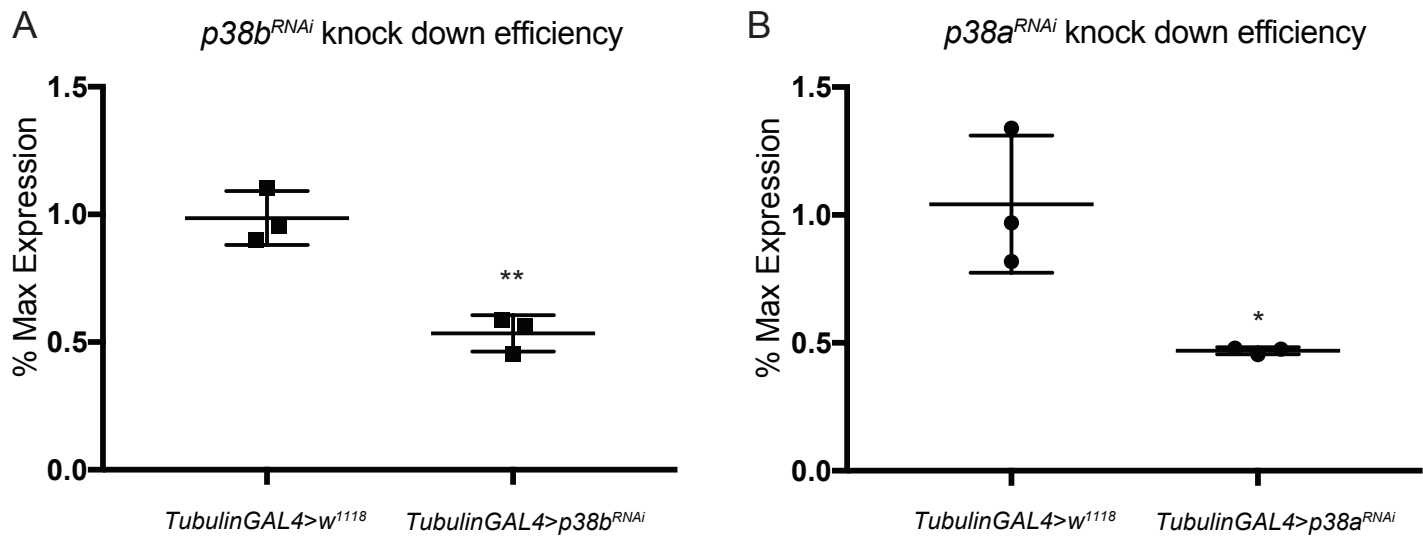

Supplement: S3 Fig — RT-PCR showing knockdown efficiencies for progeny of A) p38bRNAi fly lines crossed to Tubulin-GAL4 or B) p38aRNAi fly lines crossed to Tubulin-GAL4. Expression levels were normalized to Rp49, and are shown as % Max Expression, with maximum determined by expression of control (w1118 xTubulin-GAL4) flies. Statistical significance was determined by two-tailed unpaired t test, *p<0.05, **p<0.005. (PDF) [file ppat.1007020.s005.pdf]

S4 Fig.

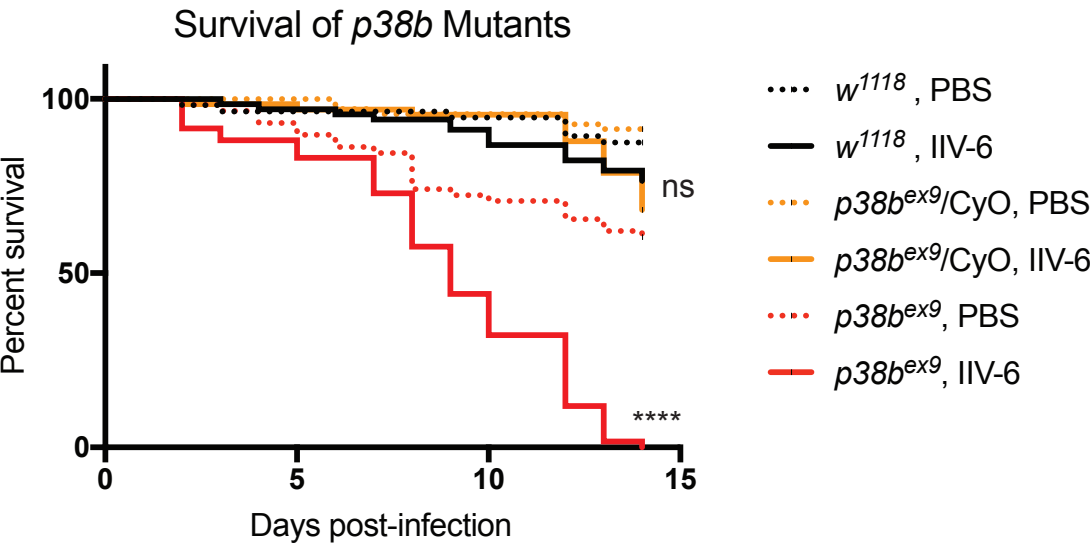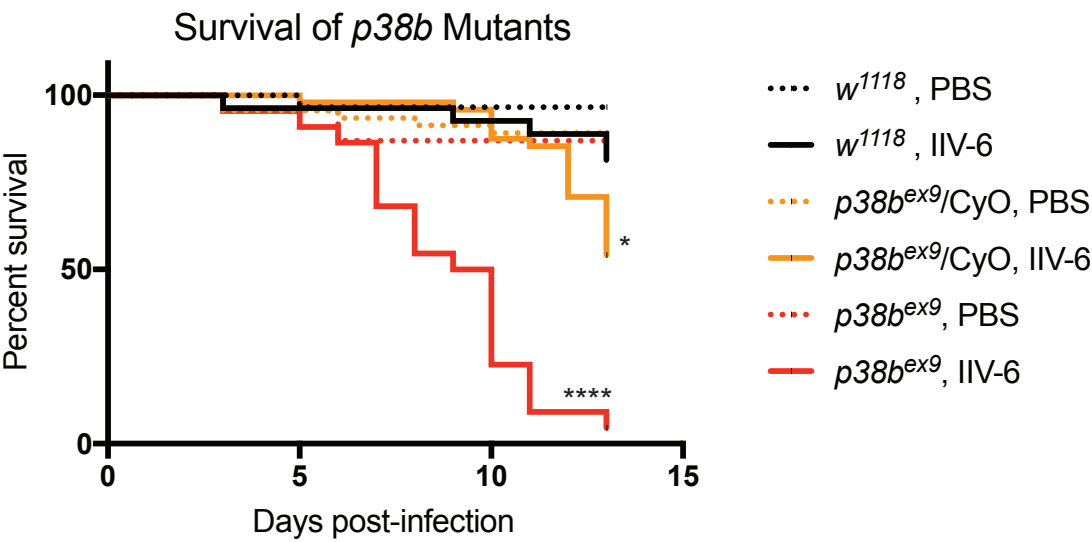

Supplement: S4 Fig — Kaplan-Meier curves showing survival of homozygous (p38bex9, red lines) or heterozygous (p38bex9/CyO, orange lines) p38b mutant flies following IIV-6 infection (solid lines) or PBS-injected controls (dotted lines) compared to control (w1118, black) flies. Statistical significance was determined by Log-rank (Mantel-Cox) test, comparing IIV-6 infected mutants to IIV-6 infected control animals, or comparing PBS-injected mutants to PBS-injected control animals. *p<0.05, ****p <0.0001. ns, not significant. (PDF) [file ppat.1007020.s006.pdf]

S5 Fig.

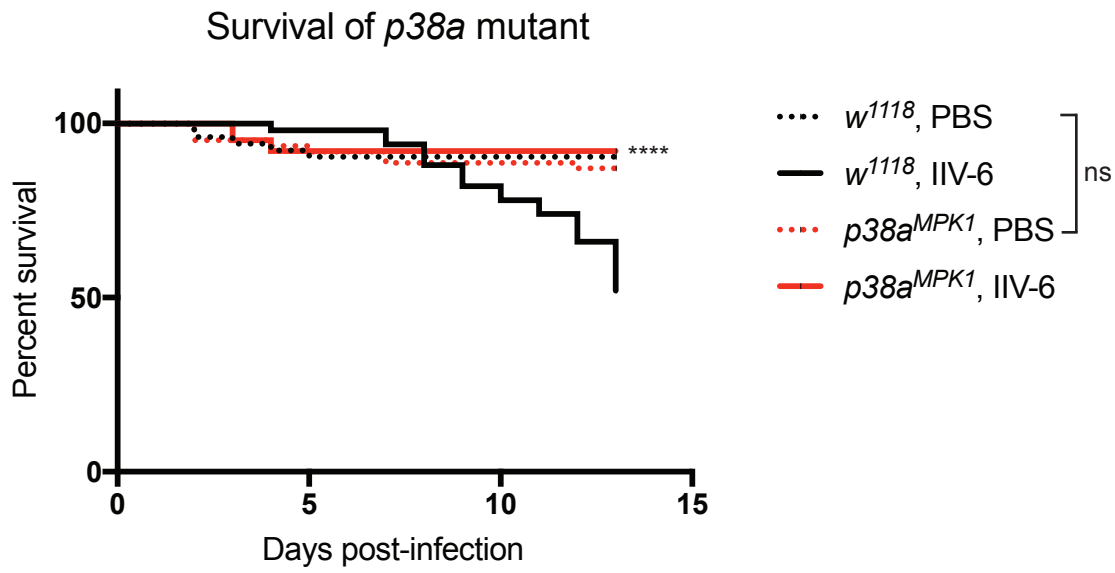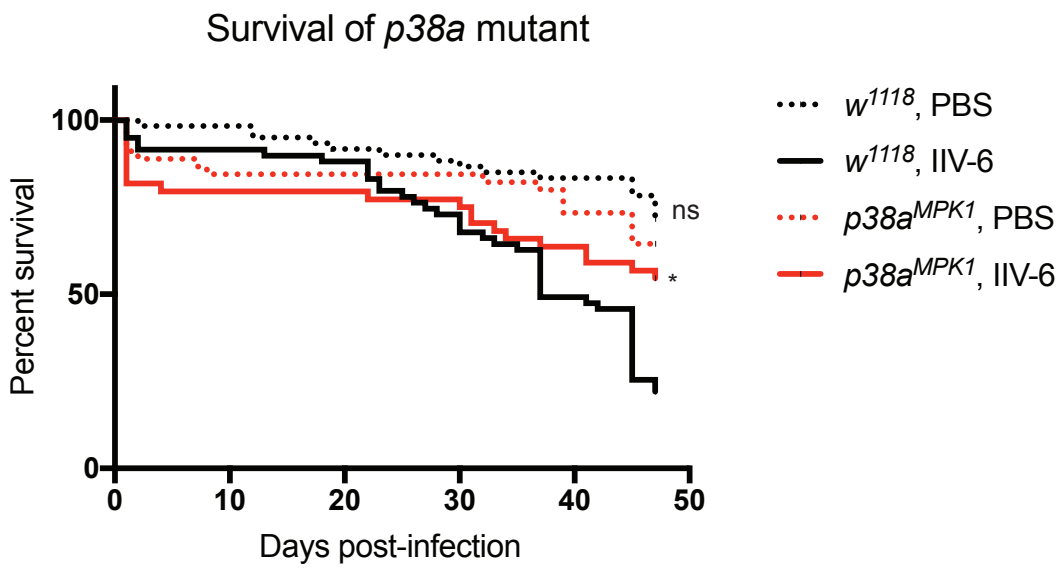

Supplement: S5 Fig — Kaplan-Meier curves showing survival of p38a mutant flies (red lines) following IIV-6 infection (solid lines) or PBS-injected controls (dotted lines) compared to control (w1118, black) flies. Statistical significance was determined by Log-rank (Mantel-Cox) test, comparing IIV-6 infected mutants to IIV-6 infected control animals, or comparing PBS-injected mutants to PBS-injected control animals. *p<0.05, ****p <0.0001. ns, not significant. (PDF) [file ppat.1007020.s007.pdf]

S6 Fig.

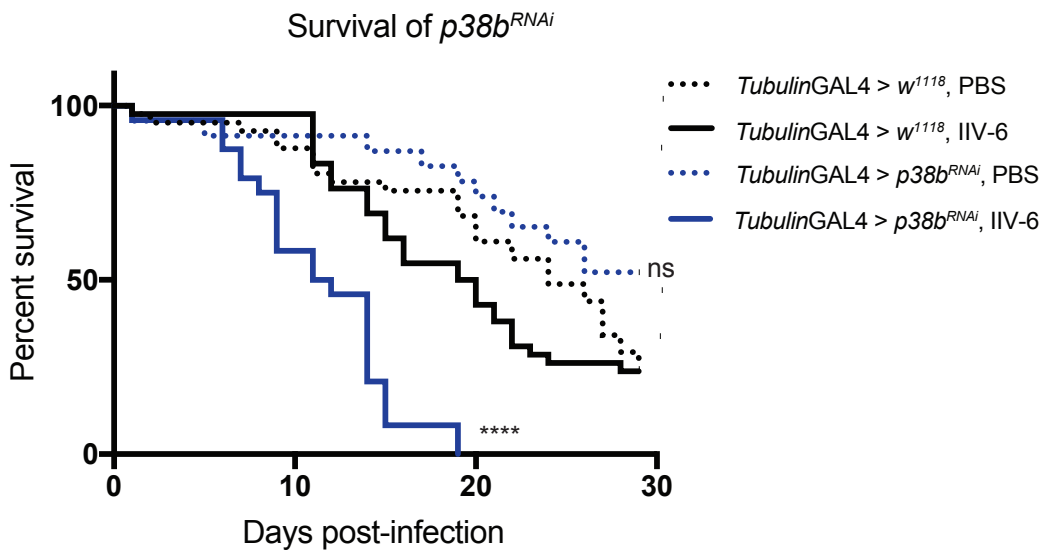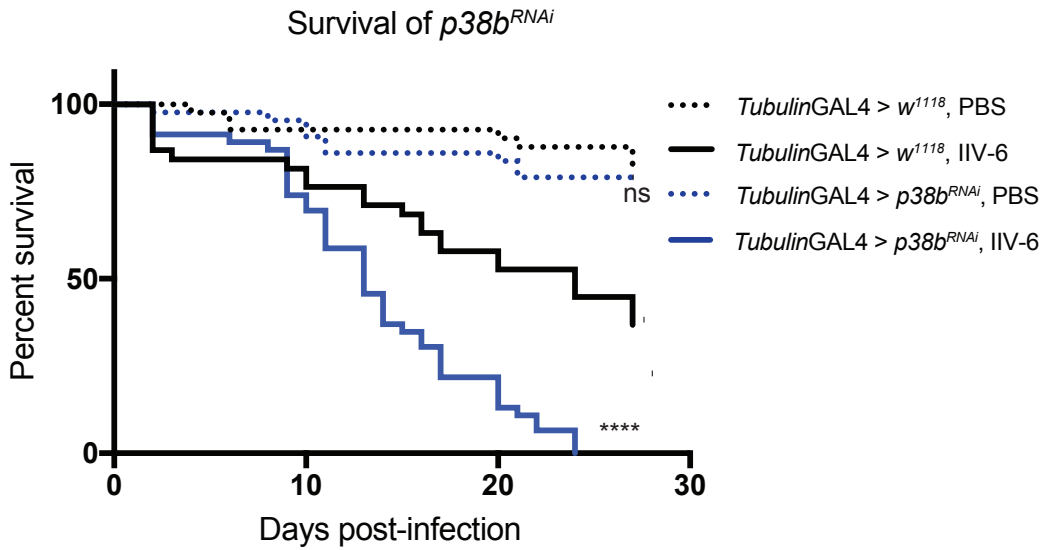

Supplement: S6 Fig — Kaplan-Meier curves showing survival of p38bRNAi expressing flies following IIV-6 infection (solid lines) or PBS-injected controls (dotted lines). UAS-p38bRNAi flies were crossed to tubulin-GAL4 for ubiquitous knock-down (blue lines), while the control was generated by w1118 crossed to tubulin-GAL4 (black lines). Statistical significance was determined by Log-rank (Mantel-Cox) test, comparing IIV-6 infected RNAi lines to IIV-6 infected control animals, or comparing PBS-injected RNAi lines to PBS-injected control lines.**** p <0.0001; ns, not significant. (PDF) [file ppat.1007020.s008.pdf]

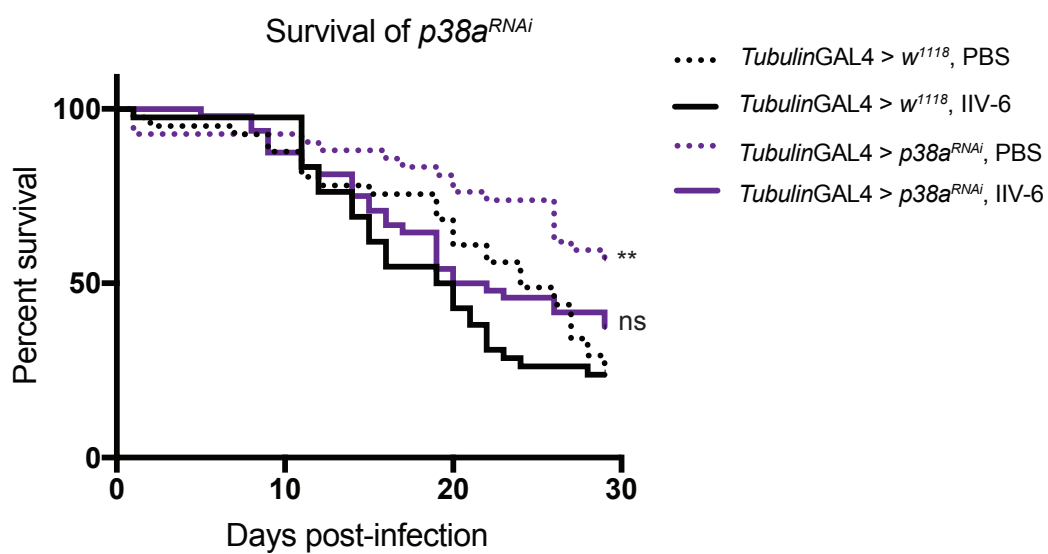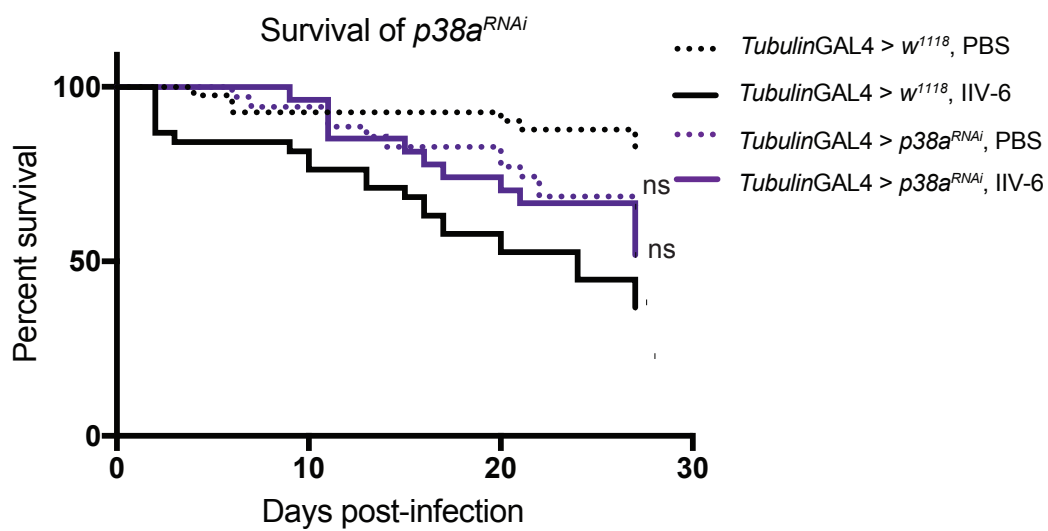

Supplement: S7 Fig — Kaplan-Meier curves showing survival of p38aRNAi expressing flies following IIV-6 infection (solid lines) or PBS-injected controls (dotted lines). UAS-p38aRNAi flies were crossed to tubulin-GAL4 for ubiquitous knock-down (purple lines), while the control was generated by w1118 crossed to tubulin-GAL4 (black lines). Statistical significance was determined by Log-rank (Mantel-Cox) test, comparing IIV-6 infected RNAi lines to IIV-6 infected control animals, or comparing PBS-injected RNAi lines to PBS-injected control lines.**** p <0.0001; ns, not significant. (PDF) [file ppat.1007020.s009.pdf]

A

Survival of *TotA* over-expressing flies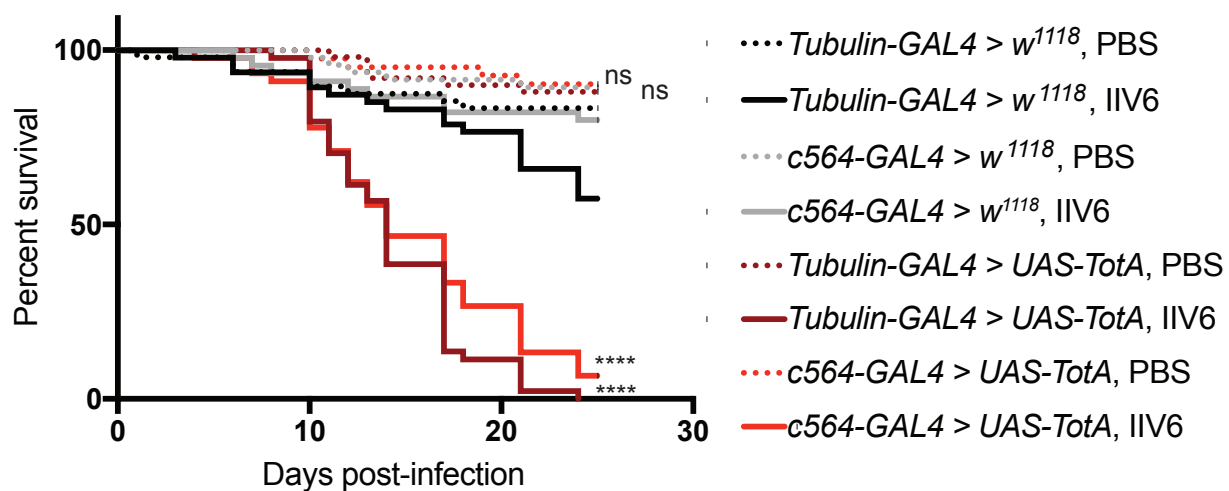

B

Viral titers of *TotA* over-expressing flies, 10dpi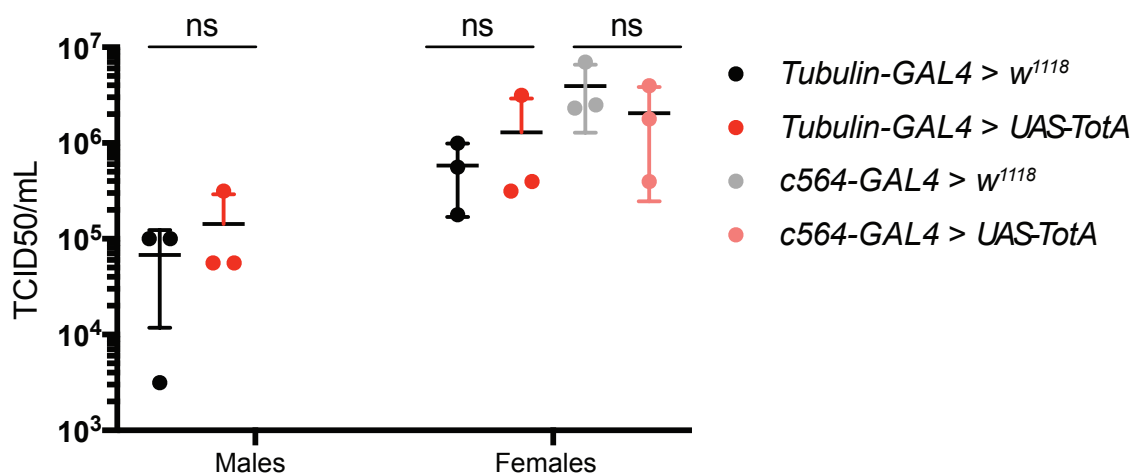

Supplement: S8 Fig — A) Kaplan-Meier curve showing survival of TotA over-expressing flies or control (w1118) flies under the control of a fat body (c564-GAL4) or ubiquitous (Tubulin-GAL4) driver, infected with IIV-6. ****p<0.0001. n>50. B) Viral loads from TotA over-expressing flies or control (w1118) flies under the control of a fat body (c564-GAL4) or ubiquitous (Tubulin-GAL4) driver infected with IIV-6 and assayed by limiting dilution (TCID50) post-infection. TCID50 was calculated using Reed-Muench method. Each data point represents 5 flies. ns, no significance. (PDF) [file ppat.1007020.s010.pdf]
